# Pirfenidone regulates seizures through the HMGB1/TLR4 axis to improve cognitive functions and modulate oxidative stress and neurotransmitters in PTZ-induced kindling in mice

**DOI:** 10.3389/fphar.2024.1528032

**Published:** 2025-01-22

**Authors:** Mansi Dahalia, Sparsh Gupta, Haya Majid, Divya Vohora, Nidhi

**Affiliations:** ^1^ Department of Pharmacology, School of Pharmaceutical Education and Research, Jamia Hamdard, New Delhi, India; ^2^ Department of Pharmacology, Vardhman Mahavir Medical College, New Delhi, India; ^3^ Department of Translational and Clinical Research, School of Chemical and Life Sciences, Jamia Hamdard, New Delhi, India

**Keywords:** anticonvulsant, cognition, epilepsy, neuroinflammation, neurotransmitters, pentylenetetrazole, pirfenidone

## Abstract

**Background:**

Epilepsy is a neurological disorder characterized by recurrent seizures due to abnormal electrical activity in the brain. Pirfenidone, an antifibrotic drug, has shown anti-inflammatory and antioxidant properties in various disease models, including neurological conditions. However, its potential anticonvulsant effects have not been thoroughly explored. This study aims to evaluate the anticonvulsant potential of pirfenidone in a pentylenetetrazol-induced kindling model of epilepsy, focusing on its effect on seizure activity, cognition, antioxidant profiles, inflammatory markers, neurotransmitter balance, liver enzyme levels, and histopathological changes.

**Methods:**

Healthy male Swiss albino mice were subjected to an acute Increasing Current Electroshock test and chronic pentylenetetrazol-kindling model. Pirfenidone was administered at doses of 100, 200, and 300 mg/kg, orally, with sodium valproate as a standard drug. Seizure severity and cognitive function were assessed in the pentylenetetrazol-kindling model, along with biochemical assays that evaluated antioxidant enzymes, inflammatory markers, neurotransmitter levels, and liver enzyme levels. Histopathological changes were also assessed in the hippocampus and cortex of experimental mice.

**Results:**

Pirfenidone at 200 mg/kg and 300 mg/kg significantly increased Seizure Threshold Current in the Increasing Current Electroshock test, indicating a protective effect against seizures. In the pentylenetetrazol-kindling model, pirfenidone delayed seizure onset and reduced severity, with the 300 mg/kg dose showing the strongest impact. Pirfenidone also demonstrated significant improvements in cognitive function, as evidenced by enhanced performance in passive avoidance and elevated plus maze tests. Antioxidant profiles showed increased levels of superoxide dismutase, catalase, and reduced glutathione, with a corresponding reduction in malondialdehyde and acetylcholinesterase levels. Pirfenidone significantly reduced pro-inflammatory cytokines including interleukin-6, interleukin-1β, transforming growth factor-β, tumor necrosis factor- α, high-mobility group box-1, and toll-like receptor-4, elevated gamma-aminobutyric acid, decreased glutamate levels, modulated aspartate aminotransferase and alanine aminotransferase levels. Histopathological analysis revealed that pirfenidone ameliorated cellular disintegration and neuronal damage in the hippocampus and cortex.

**Conclusion:**

Pirfenidone shows potential as an anticonvulsant, anti-inflammatory, hepatoprotective, and neuroprotective agent, with additional benefits in improving cognition and oxidative stress profiles in epilepsy treatment. Further studies are required to explore its long-term safety and efficacy.

## Highlights

• PFD significantly reduced key neuroinflammatory cytokines (IL-6, IL-1β, TGF-β, TNF-α, HMGB1, TLR-4) and ameliorated neuronal damage in the hippocampus and cortex, preserving structural integrity.
• PFD restored the balance between GABA and glutamate, improving inhibitory and excitatory neurotransmission, and enhanced cognitive function in PTZ-kindled mice.
• PFD increased SOD, catalase, and GSH levels while reducing MDA levels, demonstrating strong antioxidant effects and hepatoprotection by normalizing AST and ALT levels.
• PFD exhibited dose-dependent anticonvulsant effects in PTZ-kindled mice, comparable to sodium valproate (SVP), highlighting its therapeutic potential in epilepsy management.

## 1 Introduction

Epilepsy is a chronic neurological disorder affecting approximately 51 million people worldwide, with nearly 40% experiencing drug resistance (Ghaith et al., 2024; Waris et al., 2024). Beyond seizures, cognitive deficits such as memory loss and learning difficulties are common due to seizure activity, neuroinflammation, and hippocampal and cortical damage (Liu et al., 2023). Factors like seizure severity, brain damage, and side effects of anti-seizure medications worsen these impairments (Pinto et al., 2024). While older medications, including barbiturates and valproate, often impair cognition, even newer drugs like topiramate can cause long-term cognitive deficits, sometimes leading to treatment discontinuation (Tombini et al., 2023). Current anti-seizure medications (ASMs) offer symptomatic relief but fail to address the root causes of epilepsy, such as neuroinflammation, oxidative stress, and neurotransmitter imbalances (Tombini et al., 2023; Pinto et al., 2024). Their cognitive and behavioral side effects further limit long-term use, highlighting the need for therapies that not only control seizures but also target these underlying mechanisms (Pinto et al., 2024). Innovative treatments focusing on reducing neuroinflammation, mitigating oxidative stress, and restoring neurotransmitter balance are essential to improve cognitive outcomes and enhance patients’ quality of life (Khan et al., 2024; Waris et al., 2024).

Oxidative stress and impaired antioxidant defenses contribute to neuronal damage in epilepsy, characterized by increased reactive oxygen species (ROS) and lipid peroxidation (MDA), alongside decreased superoxide dismutase (SOD), catalase, and reduced glutathione (GSH) (Alam et al., 2023). Long-term antiepileptic drug use, such as valproic acid, can exacerbate oxidative stress and lipid peroxidation (Madireddy and Madireddy, 2023). Chronic oxidative damage is linked to neuronal degeneration, structural abnormalities, and cognitive impairments (Tombini et al., 2023; Khan et al., 2024; Stasenko et al., 2024). Elevated acetylcholinesterase (AChE) activity further disrupts cholinergic signaling, increasing neuronal excitability and cognitive deficits, while reducing its activity improves synaptic transmission and cognition (Akyüz et al., 2020; Komali et al., 2021; Alyami et al., 2022).

Neuroinflammation drives seizure onset and progression through astrocyte and microglial activation, releasing cytokines such as IL-6, IL-1β, and TNF-α, and disrupting the blood-brain barrier (Tavakoli et al., 2023; Tyagi et al., 2023). Key mediators of neuroinflammation include TGF-β, which regulates immune responses, and HMGB1, which activates TLR4, promoting pro-inflammatory cytokine release and neuronal hyperexcitability (Vezzani et al., 2008; Kamali et al., 2020). Imbalance of neurotransmitters, particularly gamma-aminobutyric acid (GABA) and glutamate, plays a key role in seizures. GABA, the brain’s primary inhibitory neurotransmitter, reduces neuronal excitability, while excessive glutamate release during seizures causes excitotoxicity and neuronal damage (Ivens et al., 2007; Tian et al., 2016; Nodirahon et al., 2024). Despite various ASMs targeting sodium and calcium channels, GABAergic pathways, and glutamate inhibition, many patients remain treatment-resistant, highlighting the need for novel therapeutic strategies (Imran et al., 2023; Mir et al., 2023; Löscher et al., 2020).

Pirfenidone (PFD), a pyridone-derivative compound (5-methyl-1-phenyl-2-[1H]-pyridone), is an orally administered drug approved for idiopathic pulmonary fibrosis (IPF) due to its established efficacy (Nakamura et al., 2023). Beyond fibrosis, PFD shows potential in neurological conditions like neurofibromatosis, secondary progressive multiple sclerosis, and limbic seizures (Babovic-Vuksanovic et al., 2004). Its therapeutic effects stem from modulating cytokines and growth factors such as TGF-β, TNF-α, and IL-6, reducing inflammation and immune responses (Aimo et al., 2022). PFD also inhibits lipid peroxidation in experimental models, offering protection against tissue damage (Misra and Rabideau, 2000; Salazar-Montes et al., 2008). Neuroprotective effects have been demonstrated through reduced oxidative stress and neuronal loss in seizure-related brain injuries, including kainic acid-induced excitotoxicity and traumatic brain injury models (Castro-Torres et al., 2014; 2015; Bozkurt et al., 2022). However, its direct anticonvulsant effects remain unexplored. Given its anti-inflammatory, antioxidant, and neuroprotective properties, this study aims to evaluate PFD’s anticonvulsant potential in a pentylenetetrazole (PTZ) -kindling model, focusing on oxidative stress, neuroinflammatory pathways, and key neurotransmitter systems involved in seizure development.

## 2 Material and methods

### 2.1 Animals

Healthy male Swiss albino mice weighing 25 g–30 g, were procured from the Central Animal House Facility of Jamia Hamdard, New Delhi, India. The mice were kept in polypropylene cages (43 cm × 28.6 cm × 15.5 cm) with unrestricted access to *ad libitum* water and a pellet diet. Animal care procedures adhered to the guidelines set by the Committee for Control and Supervision of Experiments on Animals (CCSEA), India, as well as the protocols established by the Institutional Animal Ethics Committee of Jamia Hamdard, New Delhi, India (173/GO/Re/2000/CPCSEA, 28 January 2000) under an approved protocol (Protocol no. 1906).

### 2.2 Materials

PFD 200 mg tablets (Pirfenex 400, Cipla Pvt. Ltd., India) and Sodium Valproate (SVP) 200 mg tablets (Valprol-CR 200, INTAS Pharmaceuticals Ltd., India) were purchased. Pentylenetetrazole (PTZ) was obtained from Sigma-Aldrich, United States. All other chemicals utilized were of analytical grade quality.

## 3 Methodology

### 3.1 Acute study: increasing current electroshock test

The mice were randomly assigned to six groups (n = 6). Group I functioned as the control, and Group II, the positive control for the ICES test, was given SVP at 200 mg/kg *p.o*., while Groups III, IV, and V received PFD at doses of 100, 200, and 300 mg/kg *p.o*., respectively. Group VI received a combination of PFD (300 mg/kg *p.o*.) and SVP (200 mg/kg p.o.). All treatments were administered 1 h before the ICES test, except Group VI, which received PFD 1 h before SVP; the ICES test was conducted 1 h after SVP. Using an electro-convulsiometer, each mouse was administered an electric shock via ear electrodes, starting with an initial current of 2 mA delivered as a single train of pulses for 0.2 s. The intensity of the current was gradually increased at a rate of 2 mA every 2 s. The seizure threshold current (STC) was determined as the current level that triggered tonic hind limb extension (HLE). If tonic HLE was not observed up to the maximum current of 30 mA, no further increase in current was applied, and this maximum value was recorded for subsequent calculations (Agarwal et al., 2013).

### 3.2 Chronic study

The experimental design involved randomly assigning animals into seven distinct groups, each comprising nine mice. The seizure scores were evaluated using a sample size of nine animals per group. Additionally, behavioral and biochemical assessments were conducted with six animals per group, while histopathological analysis was carried out using three animals per group.

The sample size was calculated using G* Power software, version 3.1. The groups were as follows:

⁃ Group I (Control): 1% carboxymethylcellulose (CMC) administered orally and normal saline administered intraperitoneally.
⁃ Group II: 1% CMC administered orally along with PTZ (25 mg/kg, *i.p.*) administered every other day.
⁃ Group III (Standard): SVP at 200 mg/kg *p.o* administered orally in addition to PTZ (25 mg/kg, *i.p.*) (Kumar et al., 2016)
⁃ Group IV: PFD at 100 mg/kg *p.o* administered orally and PTZ (25 mg/kg, *i.p.*) (Castro-Torres et al., 2014)
⁃ Group V: PFD at 200 mg/kg *p.o* administered orally along with PTZ (25 mg/kg, *i.p.*) (Liu et al., 2024)
⁃ Group VI: PFD at 300 mg/kg *p.o* administered orally along with PTZ (25 mg/kg, i.p.) (Liu and Shi, 2019)
⁃ Group VII: Combination of PFD (300 mg/kg *p.o*) and SVP (200 mg/kg *p.o*) along with PTZ (25 mg/kg, *i.p.*).

### 3.3 Drug administration protocol

PFD and SVP were weighed and suspended in 1% carboxymethyl cellulose (CMC) prepared with double-distilled water. PTZ was dissolved in normal saline (0.9% sodium chloride solution) and administered on alternate days. Control animals were given a suspension of 1% CMC in double-distilled water, administered orally once daily. The doses selected for PFD were 100, 200, and 300 mg/kg *p.o*. and were administered daily, while SVP was administered at a dose of 200 mg/kg *p.o.* Daily, 1 h before PTZ administration for 42 days. In the PTZ-kindling model, a sub-convulsant dose of PTZ (25 mg/kg, *i.p*.) was administered on alternate days, whereas treatments PFD and SVP were administered daily for 42 days. All drugs were administered in a volume of 10 mL/kg.

### 3.4 Pentylenetetrazol-kindling in mice

Following PTZ administration, the animals were monitored for 30 min to assess seizure intensity using the Racine score (Sehar et al., 2015; Khatoon et al., 2021). Seizure severity was evaluated with the following scoring system: Stage 0: no response; Stage 1: ear and facial twitches; Stage 2: myoclonic body jerks without an upright position; Stage 3: myoclonic jerks with an upright posture and bilateral forelimb clonus; Stage 4: tonic-clonic seizures; Stage 5: generalized tonic-clonic seizures with loss of postural control. Mice were deemed fully kindled after displaying a seizure score of four during three consecutive PTZ injections. The cumulative seizure score for each group was calculated at the end of the 1st, 2nd, 3rd, 4th, 5th, and 6th week. The animals remained drug-free for 3 days after the development of kindling, and then on 4th day animals were subjected to behavioral assessment followed by sacrificing them using CO_2_ chamber (Zhao et al., 2022) for biochemical estimations on 8th day.

### 3.5 Behavioral assessment

#### 3.5.1 Step down latency on passive avoidance response apparatus

The Step-Down Latency (SDL) test was conducted using a passive avoidance response apparatus to evaluate the effects of PFD on cognition. In the center of the apparatus’s grid floor, a glass Petri dish was placed upside down, creating a shock-free zone. Each mouse was positioned in this shock-free zone, and upon stepping down, it received a mild electric shock of 20 V through the grid floor. This training session lasted 1 min. After training, the time it took for the mouse to step down was recorded, with no shock administered, and this time was termed acquisition latency. The same procedure was repeated 24 h later, with the recorded time termed retention latency. Mice that did not step down within 600 s were assigned this cut-off time (Khatoon et al., 2023).

#### 3.5.2 Transfer latency on elevated plus maze apparatus

The elevated plus maze test was conducted to assess the impact of PFD on spatial learning and memory. The maze consists of four arms, two open and two closed, each measuring 16 cm in length and 5 cm in width. The closed arms are 15 cm high, extending from a central platform (5 cm × 5 cm), with the entire setup elevated 25 cm above the ground. Each mouse was initially placed at the end of an open arm. Transfer latency (TL) was defined as the time it took for a mouse to move from the open arm to one of the closed arms with all four limbs. The TL measured on the first day for each mouse was recorded as acquisition latency. If a mouse did not enter any closed arm, it was gently guided to one, and a TL of 90 s was assigned. After this, each mouse was allowed to explore the maze for an additional 2 min before being returned to its cage. Retention latency was evaluated 24 h later to assess memory retention of the task (Sehar et al., 2015).

### 3.6 Biochemical estimations

#### 3.6.1 Tissue preparation and biochemical analysis

The animals were euthanized after neurobehavioral assessment, for tissue preparation and biochemical analysis. Blood was drawn from the tail vein before euthanizing the animals, and serum was separated and preserved for measuring aspartate aminotransferase (AST) and alanine aminotransferase (ALT) levels. The separated serum was aliquoted to avoid repeated freeze-thaw cycles and stored at −80°C for further analysis. After sacrificing, the whole brain was isolated and rinsed with cold 0.9% saline, then the brain tissues hippocampus, and cerebral cortex, were excised and weighed. The tissues were snap-frozen in liquid nitrogen and stored at −80°C until biochemical and histological analyses were performed. The tissues were homogenized in 0.1 M Phosphate Buffered Saline (PBS, pH 7.4) to create a 10% homogenate while maintaining the sample on ice. The homogenates were then centrifuged at 10,000 rpm for 20 min at 4°C, and the resulting post-mitochondrial supernatant was used for the estimation of inflammatory cytokines (IL-6, IL-1β, TGF-β, TNF-α, HMGB-1, and TLR-4), neurotransmitters (GABA and glutamate), and liver enzyme levels (AST and ALT) using ELISA kits and also for the estimation of reduced GSH (Jollow et al., 1974), and antioxidant enzymes like SOD (Misra and Fridovich, 1972) and catalase (Greenwald, 2018) and AChE (Ellman et al., 1961) and MDA (Ohkawa et al., 1979). Protein estimation in hippocampus and cortex homogenates was determined by the method of Lowry et al. (1951) using bovine serum albumin as standard.

### 3.7 Estimation of oxidative stress parameters

#### 3.7.1 Estimation of lipid peroxidation

MDA, a byproduct of lipid peroxidation, was measured to assess the extent of lipid peroxidation in cortex and hippocampal tissue homogenates. This colorimetric assay relies on the reaction between thiobarbituric acid and lipid peroxides, forming a pink chromogen that can be quantified spectrophotometrically at a wavelength of 535 nm. In brief, 100 µg of cortex and hippocampal homogenate protein was mixed with 1 mL of 20% trichloroacetic acid (adjusted to pH 3.5), 1 mL of 0.67% thiobarbituric acid, and 0.1 mL of 8% sodium dodecyl sulfate (SDS). The reaction mixture was then heated in a water bath at 95°C for 1 h. After heating, the pink chromogen was extracted using a 10:1 solution of butanol and pyridine, and absorbance was measured at 535 nm against a blank. The results were calculated as nmol MDA per hour per mg protein using a molar extinction coefficient of (1.56 × 10 5 M^−1^ cm^−1^) (Ohkawa et al., 1979).

#### 3.7.2 Estimation of superoxide dismutase activity

The reaction mixture was prepared by combining 1.8 mL of glycine buffer (50 mM, pH 10.4) with 0.2 mL of phenazine methosulfate. The reaction was initiated by adding (−) epinephrine. Superoxide dismutase enzyme activity was assessed by monitoring the autoxidation of (−) epinephrine at 480 nm over a period of 3 min. Enzyme activity was expressed as nanomoles of (−) epinephrine protected from oxidation per minute per mg of protein, using a molar extinction coefficient of 4.02 × 103 M^−1^ cm^−1^ (Misra and Fridovich, 1972).

#### 3.7.3 Estimation of catalase activity

A reaction mixture of 3 mL was prepared, consisting of 2.0 mL of PBS (0.1 M, pH 7.4), 0.95 mL of hydrogen peroxide (0.019 M), and 0.05 mL of 10% phenazine methosulfate. Enzyme activity was determined by measuring the change in absorbance at 240 nm. Catalase activity was calculated as micromoles of hydrogen peroxide decomposed per minute per milligram of protein, using a molar extinction coefficient of 40 M^−1^ cm^−1^ (Greenwald, 2018).

#### 3.7.4 Estimation of reduced glutathione

Phenazine methosulphate was precipitated by mixing with 4% sulfosalicylic acid in a 1:1 ratio. The samples were then stored at 4°C for 1 h, followed by centrifugation at 4,000 rpm (5,600 g) for 15 min at 4°C. The assay mixture, totaling 3 mL, consisted of 0.4 mL of the resulting supernatant, 2.2 mL of 0.1 M sodium phosphate buffer (pH 7.4), and 0.4 mL of 5,5′-dithio-bis-2-nitrobenzoic acid (DTNB). The absorbance of the reaction product was measured at 412 nm using a spectrophotometer. The concentration of reduced glutathione (GSH) was calculated as micromoles of GSH per milligram of tissue, employing a molar extinction coefficient of 1.36 × 104 M^−1^ cm^−1^ (Jollow et al., 1974).

### 3.8 Estimation of acetylcholinesterase activity

The reaction mixture consisted of 0.2 mL of acetylthiocholine iodide (75 mM), 0.1 mL of buffered Ellman’s reagent DTNB (10 mM in 15 mM NaHCO₃), and 3 mL of PBS (25 mM, pH 7.4). This mixture was incubated at room temperature for 10 min. After incubation, 0.2 mL of tissue homogenate was added. The optical density (OD) was then measured at 412 nm within 5 min using a spectrophotometer to determine the rate of substrate hydrolysis, expressed as micromoles of substrate hydrolyzed per minute per milligram of protein (Ellman et al., 1961).

### 3.9 Estimation of neuroinflammatory markers, neurotransmitters, and liver enzymes via enzyme-linked immunosorbent assay

The levels of IL-6, IL-1β, TGF-β, TNF-α, HMGB-1, TLR-4, GABA, and glutamate in the hippocampus and cortex tissues of the experimental mice were quantitatively assessed. Additionally, serum samples were analyzed to measure AST and ALT levels. All the estimations were done using ELISA kits from KRISHGEN BioSystems, following the manufacturer’s guidelines. All assays were performed according to the manufacturer’s protocols.

### 3.10 Histopathological studies

The mice from each group were sacrificed and brains were extracted and fixed in 4% paraformaldehyde at 4°C for 4 h, then embedded in paraffin wax. Coronal and hippocampal sections (4 μm thick) were prepared and mounted on polylysine-coated slides. The Nissl Staining was performed using paraffin sections which were deparaffinized and rehydrated through a sequence of washes: two 10-min washes in xylene, two 5-min washes in 100% ethanol, a 5-min wash in 95% ethanol, a 5-min wash in 90% ethanol, a 3-min wash in 80% ethanol, a 3-min wash in 70% ethanol, and a final 5-min rinse in distilled water. Sections were then stained with 0.1% cresyl violet solution (prepared in 0.01% glacial acetic acid) at 37°C for 10 min, rinsed briefly in distilled water, and differentiated in 95% ethanol for 30 s. Neuronal damage was evaluated under a 400x microscope (Wu et al., 2021).

### 3.11 Statistical analysis

The experimental findings are presented as Mean ± Standard Error of the Mean (SEM). Data were analyzed using one-way ANOVA, followed by Tukey’s multiple comparison test except seizure score which was analyzed using two-way ANOVA, followed by Tukey’s multiple comparison test. All statistical analyses were conducted with GraphPad Prism 9 software, and a p-value of less than 0.05 was regarded as statistically significant.

## 4 Results

### 4.1 Effect of pirfenidone administration on the increasing current electroshock test in mice

In the ICES test, PFD 100 mg/kg *p.o*., did not significantly increase the STC compared to the control group. Group II, which received 200 mg/kg *p.o.* of SVP orally (acting as the positive control), demonstrated a highly significant increase in STC (p < 0.001) compared to the control group. Similarly, Groups IV and V, treated with PFD at doses of 200 mg/kg *p.o* and 300 mg/kg *p.o* respectively, showed a statistically significant increase in STC (p < 0.05 and p < 0.001, respectively) compared to the control group. This suggests that PFD at doses of 200 mg/kg *p.o* and 300 mg/kg offers protection against hind limb extension (HLE) comparable to that of SVP. Moreover, the combination treatment in Group VI (SVP + PFD 300 mg/kg) also led to a significant increase in STC compared to the control group suggesting a potential enhancement in efficacy of the combination of PFD and SVP, as shown in Table 1.

**TABLE 1:** Effect of Pirfenidone Administration on the Increasing Current Electroshock Test in mice.

Group	No. of animals	STC (mA)
CONTROL	6	17.33 ± 0.71
SVP	6	27.5 ± 1.02^a^
100 PFD	6	18.5 ± 1.78
200 PFD	6	23.66 ± 1.72^b^
300 PFD	6	26.5 ± 0.92^c^
300 PFD + SVP	6	28.66 ± 0.80^d^

Data were analyzed by one-way ANOVA followed by Tukey’s multiple comparison test (*n* = 6) and expressed as mean ± SEM., Results were considered significant with p-value ≤ 0.05 and F (5,30) = 15.

^a^ P < 0.001 Control vs. SVP.

^b^ P < 0.05 Control vs. PFD 200 mg/kg.

^c^ P < 0.001 Control vs. PFD 300 mg/kg.

^d^ P < 0.001 Control vs. SVP + PFD 300 mg/kg.

### 4.2 Effect of pirfenidone administration on pentylenetetrazol -induced kindling model in mice

Mean seizure scores were calculated by the end of each week over 6 weeks. The group treated with Vehicle + PTZ developed stage 4 seizures by the 5th week, indicating the onset of generalized tonic-clonic seizures. In the first week, all groups started from a relatively similar baseline, with the Control group showing stable, low values throughout the study period. By the second week, the PTZ + Vehicle group began to deviate significantly from the Control group (p < 0.001), showing a sharp upward trend, indicating the detrimental effect of PTZ treatment. In contrast, treatment groups demonstrated varying degrees of reduction in the measured parameter compared to the PTZ + Vehicle group (p < 0.001).

**FIGURE 1:**
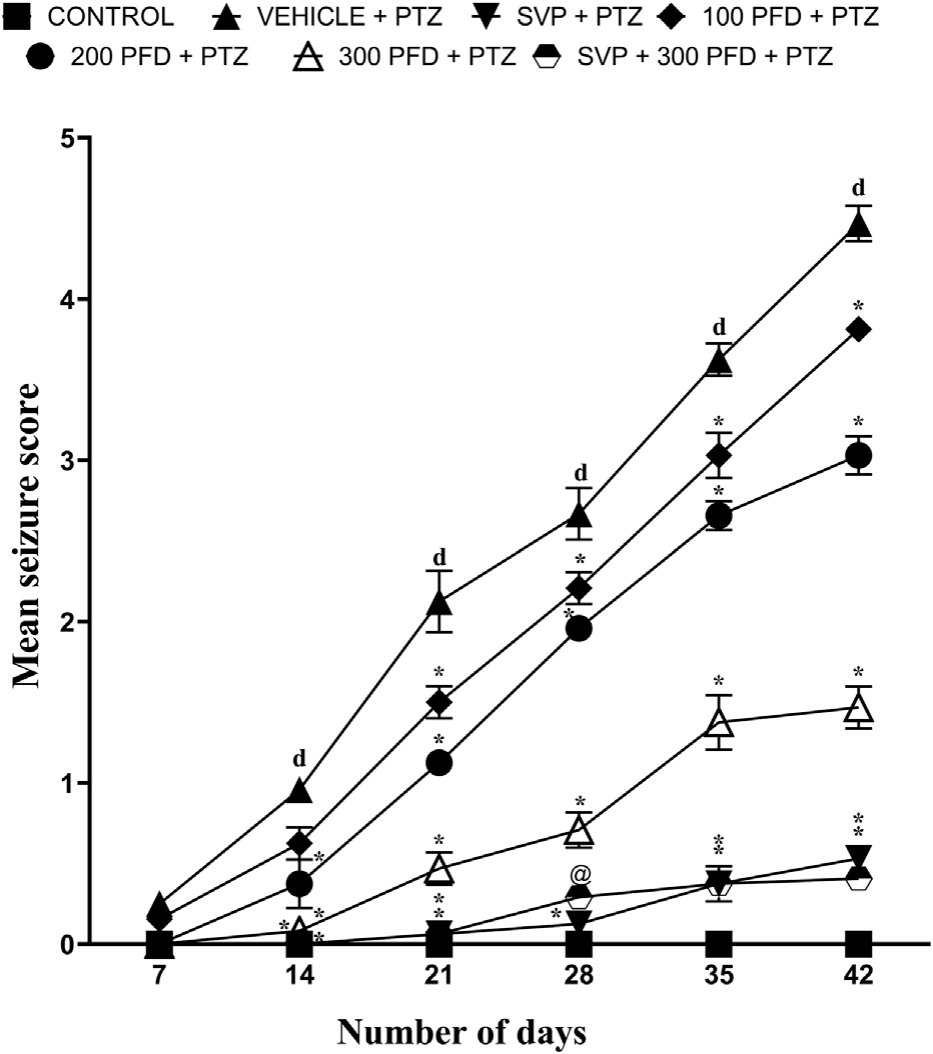
The effect of Pirfenidone administration on mean seizure scores in PTZ-induced kindling in mice. Data were analyzed by two-way ANOVA followed by Tukey’s multiple comparison test and expressed as mean ± SEM (*n* = 9). Results were considered significant with p-value ≤ 0.05 and F values F (30,294) = 57.8, F (5,294) = 523 and F (6,294) = 712; dp < 0.001 Control vs. PTZ + vehicle, *p < 0.001 PTZ + vehicle vs. SVP + PTZ; PFD 100 + PTZ, PFD 200 + PTZ, PFD 300 + PTZ, PFD 300 + SVP + PTZ, and @p < 0.01 PTZ + vehicle vs. SVP + PFD 300 + PTZ.

By the third week, the differences became more pronounced, with PFD 200 mg/kg and PFD 300 mg/kg and combination treatments showing a substantial reduction (p < 0.001). The groups receiving PFD 300 + SVP + PTZ demonstrated the most effective reduction, suggesting a possible dose-response relationship and synergistic effect. During the fourth, fifth and sixth weeks, the treatment groups maintained their respective trajectories (p < 0.001) (Figure 1).

### 4.3 Effect of pirfenidone administration on transfer latency in pentylenetetrazol-induced kindling model in mice

**FIGURE 2:**
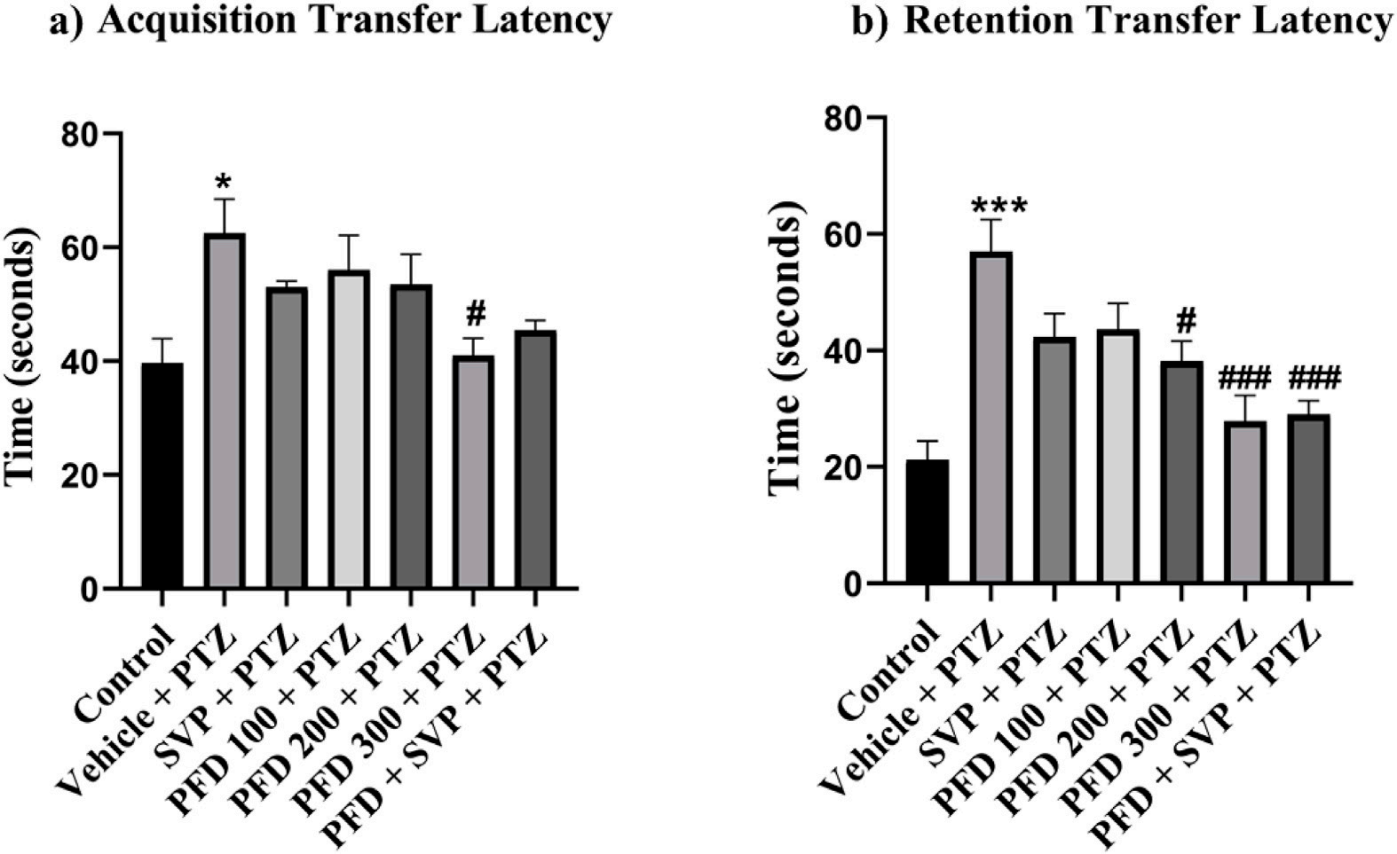
Effect of Pirfenidone administration on the transfer latency of the PTZ-induced kindling in mice. Data were analyzed by one-way ANOVA followed by Tukey’s multiple comparison tests (*n* = 6) and expressed as mean ± SEM, **(A)** Acquisition Transfer Latency F (6,35) = 3.75, **(B)** Retention Transfer latency F (6,35) = 9.01 *p < 0.05 Control vs. PTZ + vehicle, ***p < 0.001 Control vs. PTZ + vehicle, #p < 0.05 PTZ + vehicle vs. PFD200 + PTZ, PFD 300 + PTZ, ###p < 0.001 PTZ + vehicle vs. PFD300, PFD300 + SVP + PTZ.

In the elevated plus maze experiment, the vehicle + PTZ group showed an increased exploration time in the closed arms compared to the control group, indicated by a significant rise in acquisition latency (p < 0.05) and retention latency (p < 0.001). In contrast, a notable decrease in retention latency was observed in the PFD 200 + PTZ (p < 0.05), PFD 300 + PTZ (p < 0.001), and PFD 300 + SVP + PTZ (p < 0.001) groups compared to the vehicle + PTZ group. Additionally, only the PFD 300 + PTZ group demonstrated a significant reduction in acquisition latency (p < 0.05) (Figure 2).

### 4.4 Effect of pirfenidone administration on the step-down latency in pentylenetetrazol-induced kindling model in mice

**FIGURE 3:**
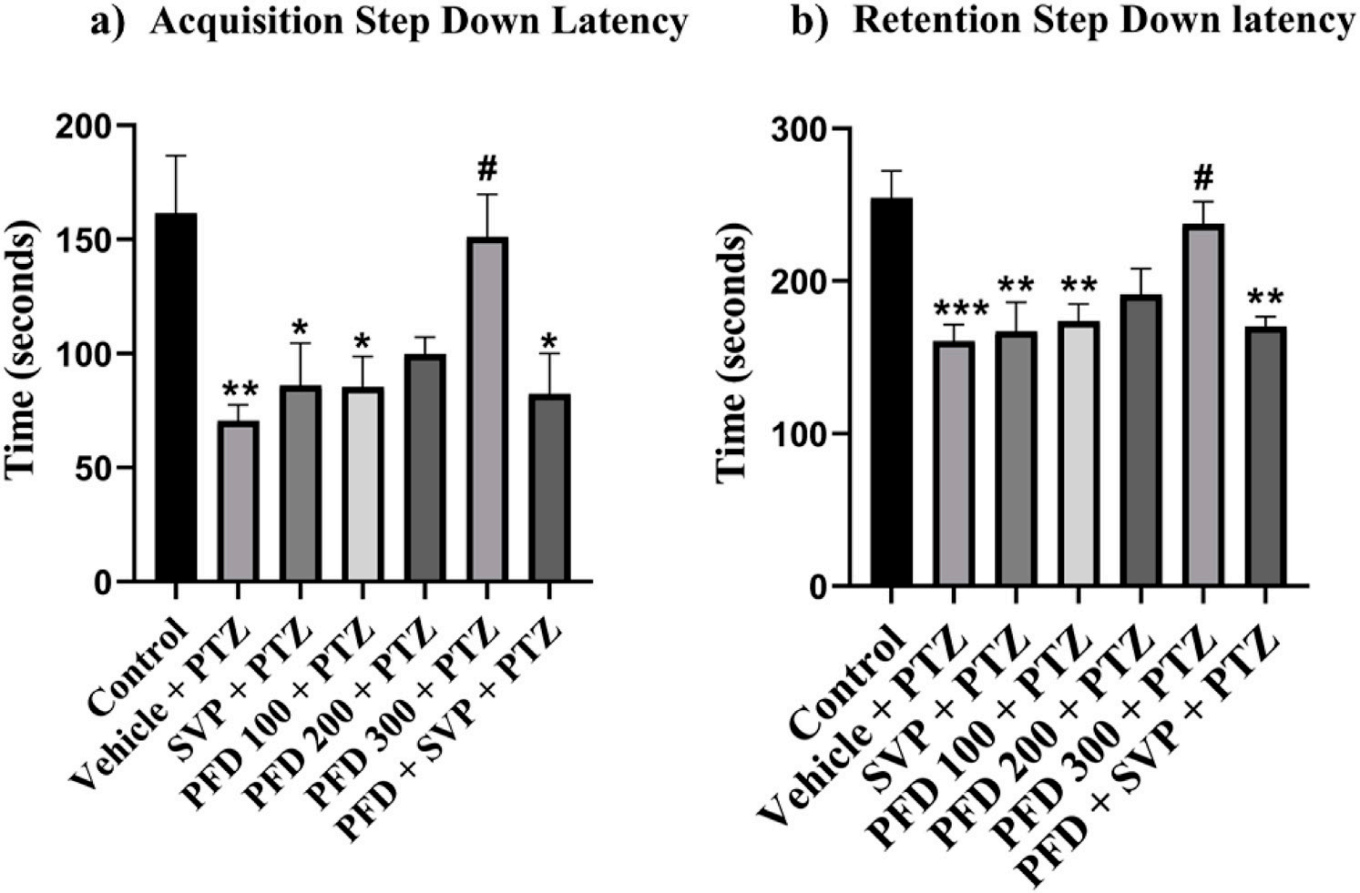
Effect of Pirfenidone administration on the step-down latency of the PTZ-induced kindling in mice. Data were analyzed by one-way ANOVA followed by Tukey’s multiple comparison tests (*n* = 6) and expressed as mean ± SEM, **(A)** Acquisition Step Down Latency F (6,35) = 4.72, **(B)** Retention step down latency F (6,35) = 6.71 **p < 0.01 Control vs. PTZ + vehicle, **p < 0.05 Control vs. PTZ + vehicle, PFD 100 + PTZ, SVP + PTZ, PFD300 + SVP + PTZ, ***p < 0.001 Control vs. PTZ + vehicle, #p < 0.05 PTZ + vehicle vs. PFD 300 + PTZ.

In the passive avoidance response test, the vehicle + PTZ group exhibited a significant reduction (p < 0.01) in both acquisition and retention latency compared to the control group, indicating impaired cognition. Similarly, the VPA + PTZ, PFD 100 + PTZ, and PFD 300 + VPA + PTZ groups also demonstrated a significant decrease (p < 0.05) in acquisition latency, with the VPA + PTZ and PFD 100 + PTZ groups also showing a significant reduction (p < 0.01) in retention latency, further suggesting cognitive impairment. However, in the PFD 300 + PTZ group, there was a significant improvement (p < 0.05) in memory function, as evidenced by a notable increase in both acquisition and retention latency compared to the vehicle + PTZ group. Mice treated with PFD displayed preserved memory function, as indicated by their increased latency to step down from the platform (Figure 3).

### 4.5 Effect of pirfenidone administration on lipid peroxidation level in pentylenetetrazol-induced kindling model in mice

**FIGURE 4:**
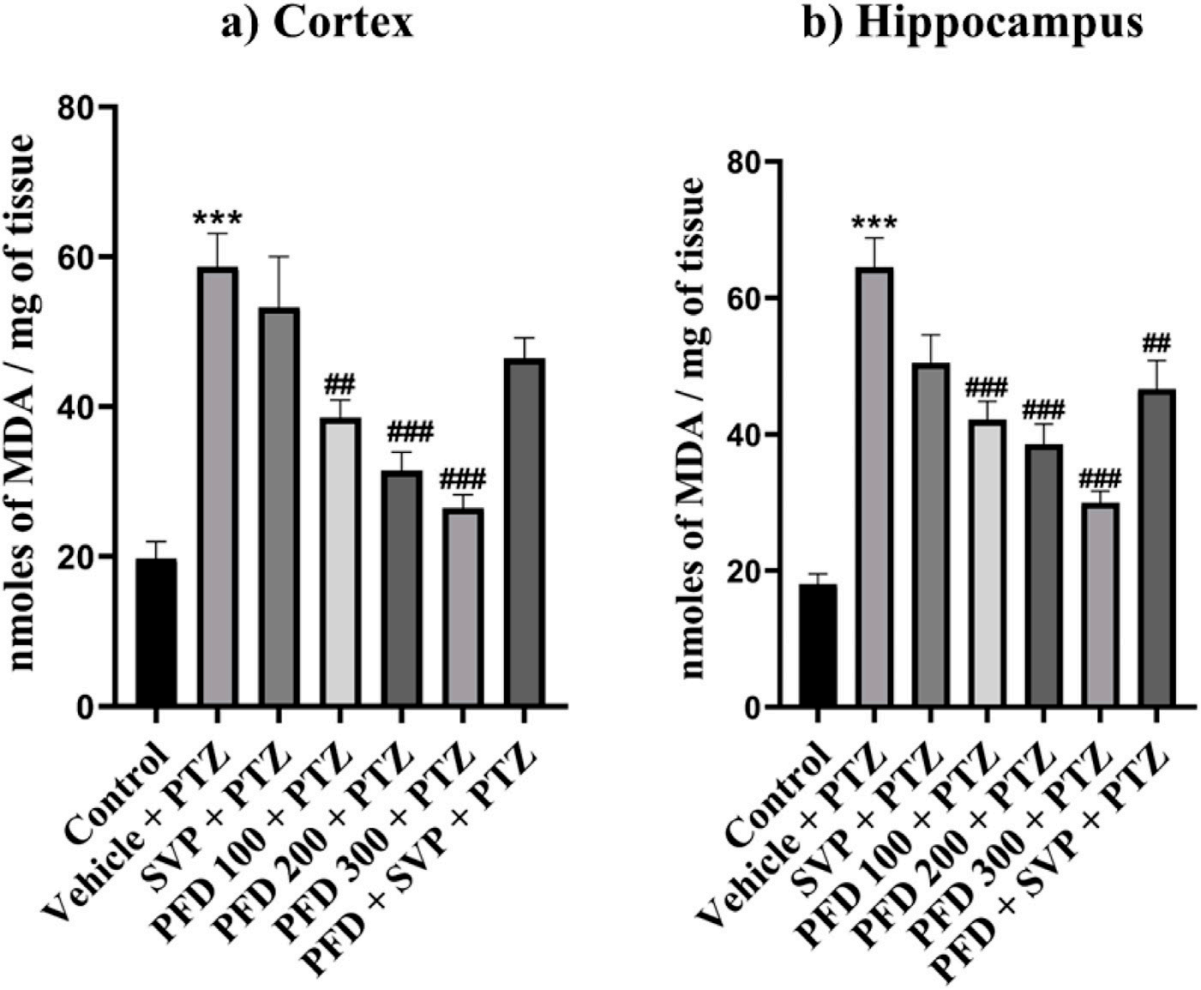
Effect of PFD administration on Malondialdehyde levels of the PTZ-induced kindling in mice. Data were analyzed by one-way ANOVA followed by Tukey’s multiple comparison tests (*n* = 6) and expressed as mean ± SEM, MDA levels in **(A)** Cortex F (6,35) = 15.5 and **(B)** Hippocampus F (6,35) = 20.9, ***p < 0.001 Control vs. PTZ + vehicle, ##p < 0.01 PTZ + vehicle vs. PFD 100 + PTZ, PFD300 + SVP + PTZ, ###p < 0.001 PTZ + vehicle vs. PFD 100 + PTZ, PFD200 + PTZ, PFD 300 + PTZ.

The MDA level was significantly elevated (p < 0.001) in the vehicle + PTZ group compared to the control group in both hippocampus and cortex. PFD 100 + PTZ (p < 0.01), PFD 200 + PTZ (p < 0.001), and PFD 300 + PTZ (p < 0.001) showed significant reduction MDA levels in cortex, similarly, all groups PFD 100 + PTZ (p < 0.001), PFD 200 + PTZ (p < 0.001), PFD 300 + PTZ (p < 0.001) and also PFD 300 + SVP + PTZ showed significant reduction MDA levels in Hippocampus (Figure 4).

### 4.6 Effect of pirfenidone administration on antioxidant profile in pentylenetetrazol-induced kindling model in mice

**FIGURE 5:**
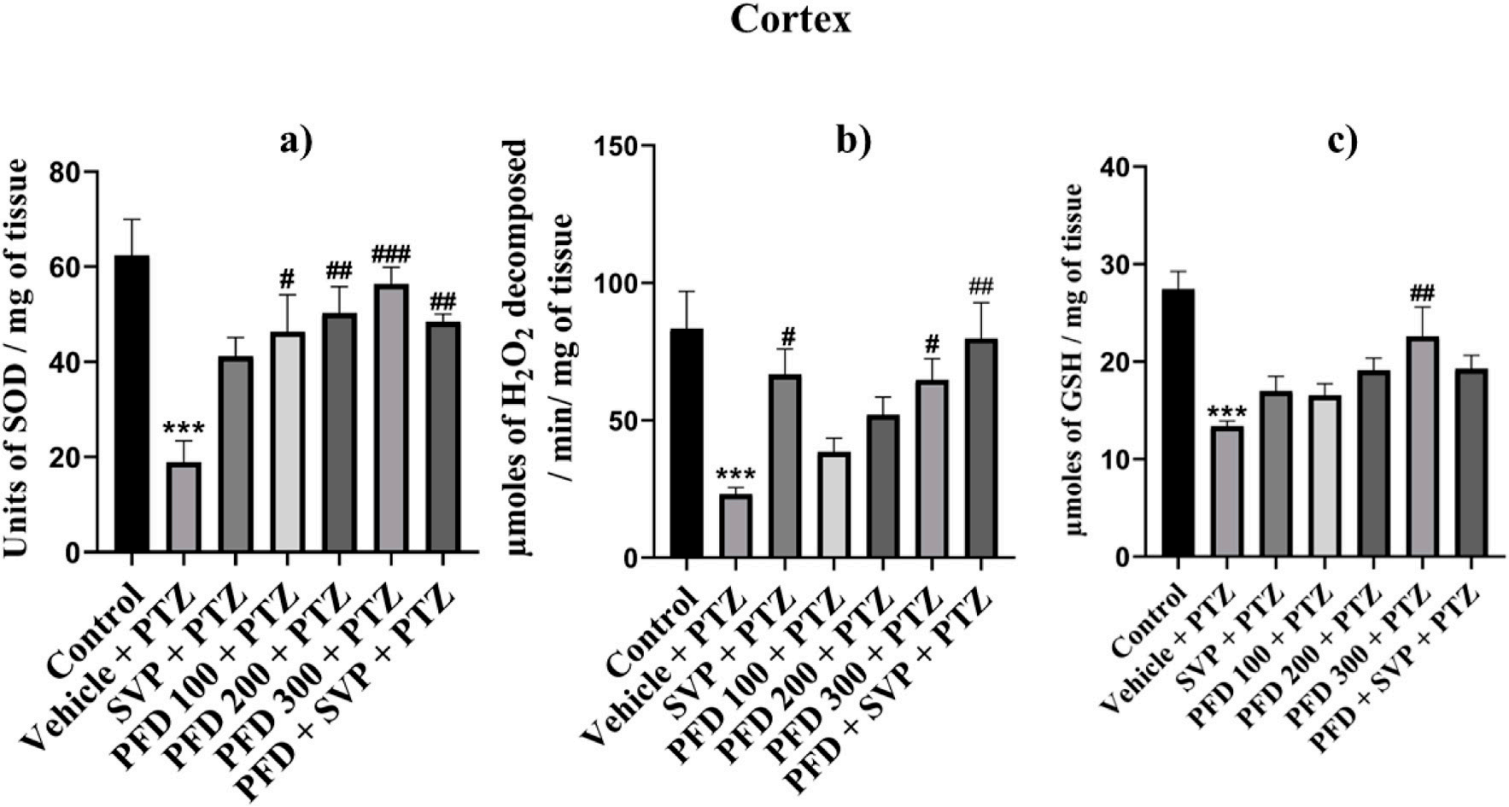
Effect of Pirfenidone administration on Superoxide dismutase, Catalase, and reduced Glutathione levels cortex in PTZ-induced kindling in mice. Data were analyzed by one-way ANOVA followed by Tukey’s multiple comparison tests (*n* = 6) and expressed as mean ± SEM, **(A)** SOD levels in Cortex F (6,35) = 6.80, ***p < 0.001 Control vs. PTZ + vehicle, #p < 0.05 PTZ + vehicle vs. PFD100 + PTZ, ##p < 0.05 PTZ + vehicle vs. PFD200 + PTZ, PFD 300 + SVP + PTZ, ###p < 0.001 PTZ + vehicle vs. PFD300 + PTZ. **(B)** Catalase levels in Cortex F (6,35) = 5.89, ***p < 0.001 Control vs. PTZ + vehicle, #p < 0.05 PTZ + vehicle vs. SVP + PTZ, PFD300 + PTZ, ##p < 0.05 PTZ + vehicle vs. PFD 300 + SVP + PTZ. **(C)** GSH levels in Cortex F (6,35) = 7.41, ***p < 0.001 Control vs. PTZ + vehicle, ##p < 0.01 PTZ + vehicle vs. PFD300 + PTZ.

**FIGURE 6:**
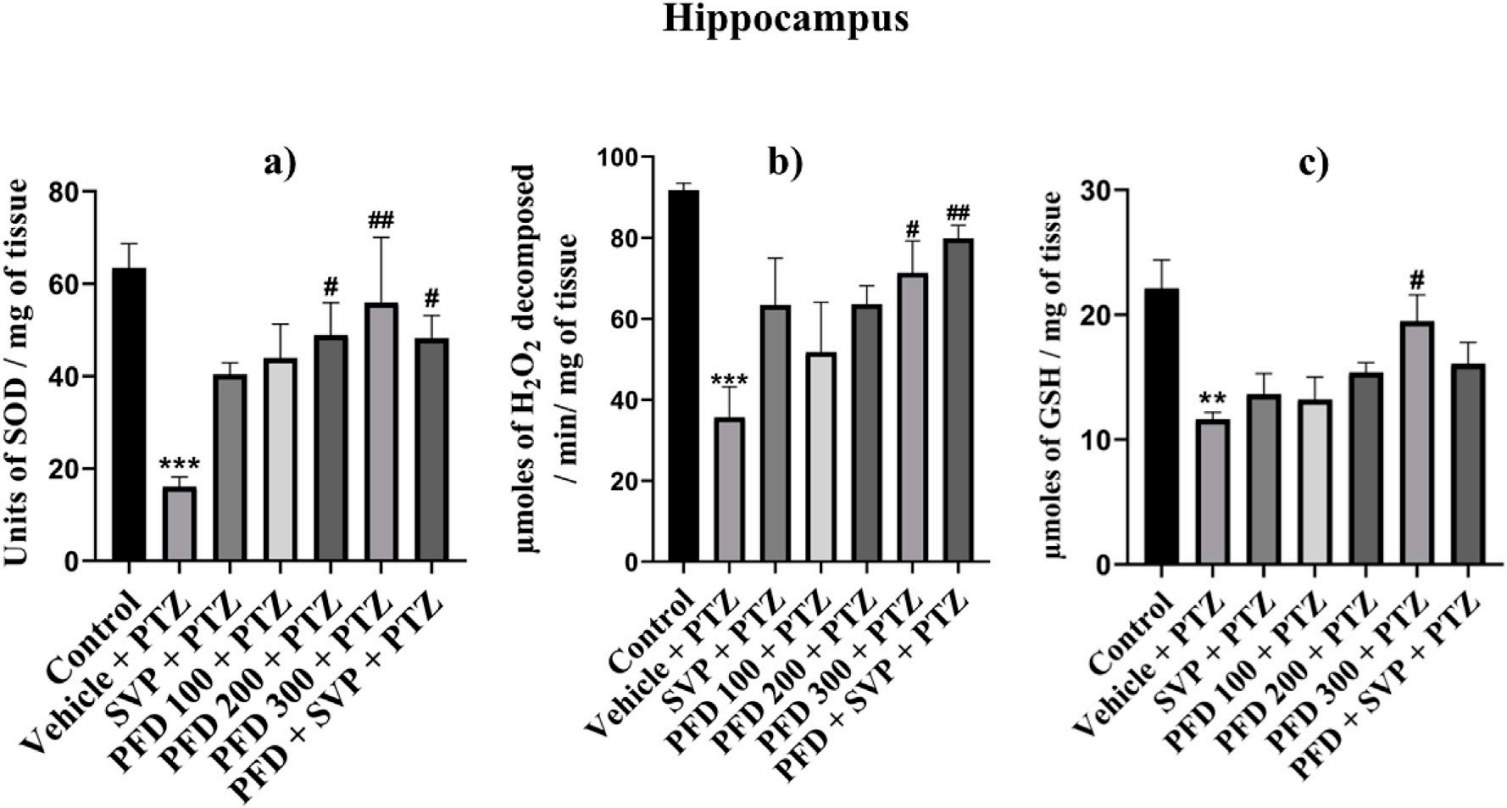
Effect of Pirfenidone administration on Superoxide dismutase, Catalase, and reduced Glutathione levels in hippocampus PTZ-induced kindling in mice. Data were analyzed by one-way ANOVA followed by Tukey’s multiple comparison tests (*n* = 6) and expressed as mean ± SEM, **(A)** SOD levels in Hippocampus F (6,35) = 4.30,***p < 0.001 Control vs. PTZ + vehicle, #p < 0.05 PTZ + vehicle vs. PFD200 + PTZ, PFD 300 + SVP + PTZ, ##p < 0.05 PTZ + vehicle vs. PFD300 + PTZ, **(B)** Catalase levels in Hippocampus F (6,35) = 5.37, ***p < 0.001 Control vs. PTZ + vehicle, #p < 0.05 PTZ + vehicle vs. PFD300 + PTZ, ##p < 0.05 PTZ + vehicle vs. PFD 300 + SVP + PTZ. **(C)** GSH levels in Hippocampus F (6,35) = 4.95, **p < 0.01 Control vs. PTZ + vehicle, #p < 0.05 PTZ + vehicle vs. PFD300 + PTZ.

The antioxidant enzymes SOD, Catalase, and GSH levels were significantly reduced (p < 0.001) in the Vehicle + PTZ group compared to the control group in the hippocampus and cortex, with the Vehicle + PTZ group also showing significant reduction (p < 0.01) in reduced GSH levels compared to the control group in the hippocampus. SOD levels were significantly improved by PFD 100 + PTZ (p < 0.05), PFD 200 + PTZ (p < 0.01), PFD 300 + PTZ (p < 0.001) and PFD 300 + SVP + PTZ (p < 0.01) in cortex, whereas in hippocampus groups PFD 200 + PTZ (p < 0.05), PFD 300 + PTZ (p < 0.01) and PFD 300 + SVP + PTZ (p < 0.05) showed significant increase in SOD levels. The catalase activity by PFD 300 + PTZ (p < 0.05) and PFD 300 + SVP + PTZ (p < 0.01) compared to vehicle + PTZ group in both hippocampus and cortex, but in cortex SVP + PTZ (p < 0.05) group also showed significant rise compared to Vehicle + PTZ group. The GSH levels were significantly raised by the PFD 300 + PTZ group compared to the Vehicle + PTZ group in the cortex (p < 0.01) and hippocampus (p < 0.05) (Figures 5, 6).

### 4.7 Effect of pirfenidone administration on acetylcholine esterase activity in pentylenetetrazol-induced kindling model in mice

**FIGURE 7:**
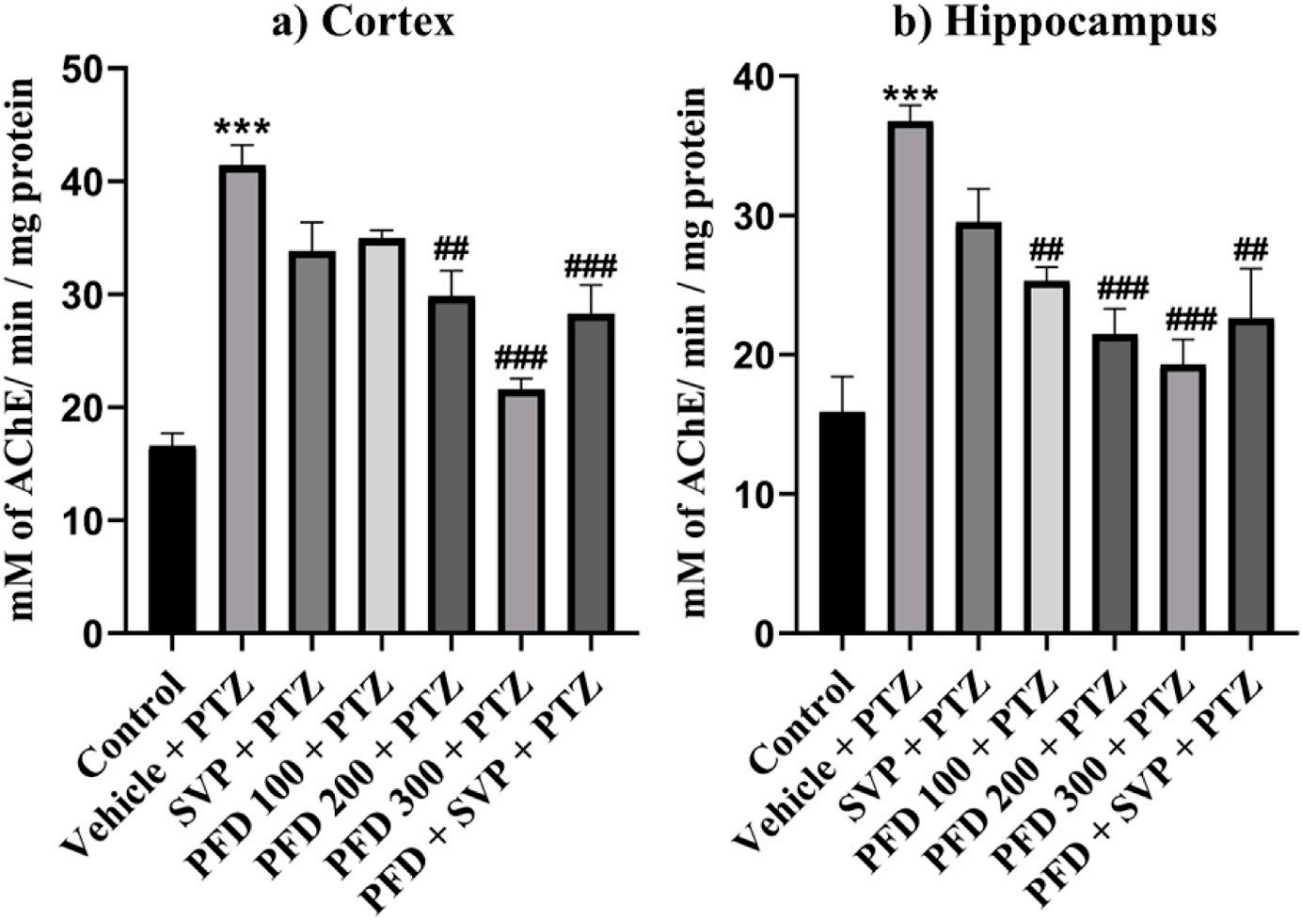
Effect of Pirfenidone on Acetylcholine estrase levels in PTZ-induced kindled mice. Data were analyzed by one-way ANOVA followed by Tukey’s multiple comparison tests (*n* = 6) and expressed as mean ± SEM, AChE levels in **(A)** Cortex F (6,35) = 20.6 and **(B)** Hippocampus F (6,35) = 10.1, ***p < 0.001 Control vs. PTZ + vehicle, #p < 0.05 PTZ + vehicle vs. PFD100 + PTZ, ##p < 0.05 PTZ + vehicle vs. PFD200 + PTZ, PFD 300 + SVP + PTZ, ###p < 0.001 PTZ + vehicle vs. PFD200 + PTZ, PFD 300 + PTZ, PFD 300 + SVP + PTZ.

The acetylcholine esterase enzyme level was significantly increased (p < 0.001) in the vehicle + PTZ group compared to the control group in both hippocampus and cortex. In the hippocampus, all the groups PFD 100 + PTZ (p < 0.05), PFD 200 + PTZ (p < 0.001), PFD 300 + PTZ (p < 0.001), and PFD 300 + SVP + PTZ (p < 0.01) showed significant decrease in AChE levels compared to vehicle + PTZ group. In the cortex, PFD 200 + PTZ (p < 0.01), PFD 300 + PTZ (p < 0.001), and PFD 300 + SVP + PTZ (p < 0.001) showed a significant decrease in AChE levels compared to vehicle + PTZ group (Figure 7).

### 4.8 Effect of pirfenidone administration on neuroinflammatory markers in the hippocampus and cortex of pentylenetetrazol-induced kindling model in mice

**FIGURE 8:**
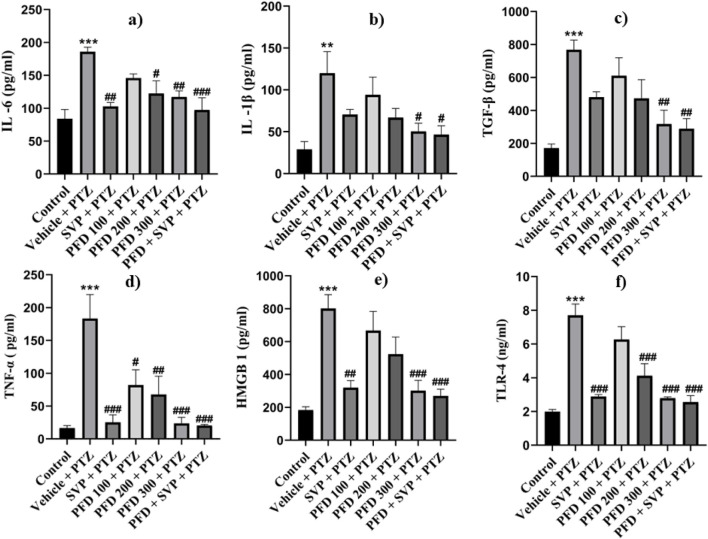
Effect of PFD on levels of IL-6, IL-1β, TNF-α, TGF-β, HMGB-1, and TLR-4 in the Cortex of the PTZ-induced kindled mice. Data were analyzed by one-way ANOVA followed by Tukey’s multiple comparison tests (*n* = 6) and expressed as mean ± SEM, In cortex **(A)** IL-6 F (6,35) = 7.34 ***p < 0.001 Control vs. PTZ + vehicle, #p < 0.05 PTZ + vehicle vs. PFD200 + PTZ, ##p < 0.01 PTZ + vehicle vs. SVP + PTZ, PFD300 + PTZ, ###p < 0.001 PTZ + vehicle vs. PFD 300 + SVP + PTZ. **(B)** IL-1β F (6,35) = 4.27, **p < 0.01 Control vs. PTZ + vehicle, #p < 0.05 PTZ + vehicle vs. PFD100 + PTZ, PFD 300 + SVP + PTZ, **(C)** TNF-ά F (6,35) = 8.87, ***p < 0.001 Control vs. PTZ + vehicle, #p < 0.05 PTZ + vehicle vs. PFD100 + PTZ, ##p < 0.01 PTZ + vehicle vs. PFD 200 + PTZ and ###p < 0.001 PTZ + vehicle vs. SVP + PTZ, PFD300 + PTZ, PFD 300 + SVP + PTZ. **(D)** TGF-β, F (6,35) = 7.15, ***p < 0.001 Control vs. PTZ + vehicle, ##p < 0.01 PTZ + vehicle vs. PFD300 + PTZ, PFD 300 + SVP + PTZ. **(E)** HMGB1, F (6,35) = 9.39, ***p < 0.001 Control vs. PTZ + vehicle, ##p < 0.01 PTZ + vehicle vs. SVP + PTZ, ###p < 0.001 PTZ + vehicle vs. PFD300 + PTZ, PFD 300 + SVP + PTZ. **(F)** TLR4, F (6,35) = 18.9, ***p < 0.001 Control vs. PTZ + vehicle, ###p < 0.001 PTZ + vehicle vs. SVP + PTZ, PFD 200+ PTZ, PFD 300 + PTZ, PFD300 +SVP + PTZ.

**FIGURE 9:**
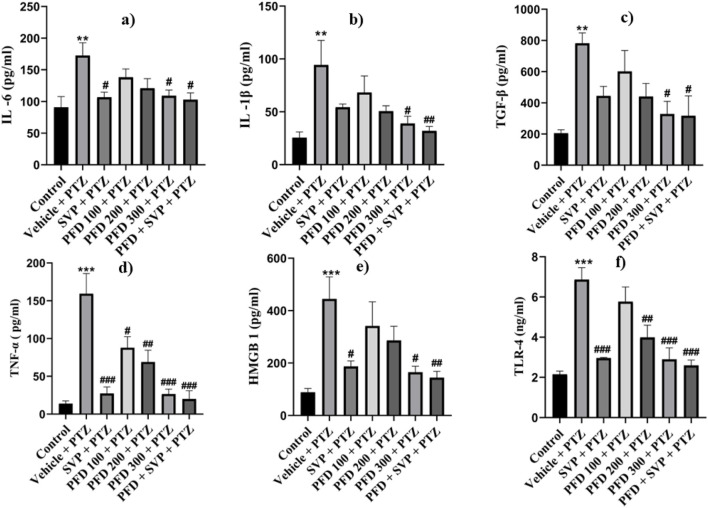
Effect of PFD on levels of IL-6, IL-1β, TNF-α,TGF-β, HMGB-1, and TLR-4 in the Hippocampus of the PTZ-induced kindled mice.Data were analyzed by one-way ANOVA followed by Tukey’s multiple comparison tests (*n* = 6) and expressed as mean ± SEM, In Hippocampus **(A)** IL-6, F (6,35) = 3.96 **p < 0.01 Control vs. PTZ + vehicle #p < 0.05 PTZ + vehicle vs. SVP + PTZ, PFD 300 + PTZ, PFD 300 + SVP + PTZ, **(B)** IL-1β F (6,35) = 4.29 **p < 0.01 Control vs. PTZ + vehicle, #p < 0.05 PTZ + vehicle vs. PFD300 + PTZ, ##p < 0.01 PTZ + vehicle vs. PFD 300 + SVP + PTZ. **(C)** TNF-&aacgr; F (6,35) = 13.8, ***p < 0.001 Control vs. PTZ + vehicle, #p < 0.05 PTZ + vehicle vs. PFD100 + PTZ, ##p < 0.01 PTZ + vehicle vs. PFD200 + PTZ, ###p < 0.001 PTZ + vehicle vs. SVP + PTZ, PFD 300 + PTZ, PFD 300 + SVP + PTZ. **(D)** TGF-β, F (6,35) = 4.70 **p < 0.01 Control vs. PTZ + vehicle, #p < 0.05 PTZ + vehicle vs. PFD300 + PTZ, SVP + PTZ, PFD 300 + SVP + PTZ. **(E)** HMGB1, F (6,35) = 5.47, ***p < 0.001 Control vs. PTZ + vehicle, #p < 0.05 PTZ + vehicle vs. PFD300 + PTZ, SVP + PTZ, ##p < 0.01 PTZ + vehicle vs. PFD 300 + SVP + PTZ. **(F)** TLR4, F (6,35) = 13.2, ##p < 0.01 PTZ + vehicle vs. PFD 200+ PTZ, ###p < 0.001 PTZ + vehicle vs. SVP + PTZ, PFD 300 + PTZ, PFD300 +SVP + PTZ.

The concentrations of inflammatory cytokines in the hippocampus and cortex of the experimental animals were evaluated using ELISA kits. In the Vehicle + PTZ group, there was a significant increase in the concentrations of IL-6 (p < 0.01), IL-1β (p < 0.01), TGF-β (p < 0.01), TNF-α (p < 0.001), HMGB1 (p < 0.001), and TLR-4 (p < 0.001) in both the hippocampus and cortex as compared to the control group. The PTZ-treated group did not show a significant reduction in the levels of IL-6, IL-1β, TGF-β, HMGB1, and TLR-4 in the hippocampus and cortex, except for TNF-α, which was significantly reduced (p < 0.05) in both regions. In contrast, all the mentioned cytokine levels were significantly reduced in the hippocampus and cortex tissues of the PFD 300 mg/kg + PTZ and PFD + SVP + PTZ groups compared to the Vehicle + PTZ group. In the PFD 200 mg/kg + PTZ group, the levels of TNF-α and TLR-4 were significantly decreased in both the hippocampus (p < 0.01) and cortex (p < 0.01, p < 0.001, respectively) of kindled mice when compared with the Vehicle + PTZ group. However, IL-1β levels were not significantly decreased in the hippocampus and cortex of the SVP + PTZ group compared to the Vehicle + PTZ group. Nevertheless, the concentrations of IL-6 (p < 0.05, p < 0.01), TNF-α (p < 0.001), HMGB1 (p < 0.05, p < 0.01), and TLR-4 (p < 0.001) were significantly decreased in the hippocampus and cortex of the SVP + PTZ group (Figures 8, 9).

### 4.9 Effects of pirfenidone administration on neurotransmitters levels in pentylenetetrazol-induced kindling in mice

**FIGURE 10:**
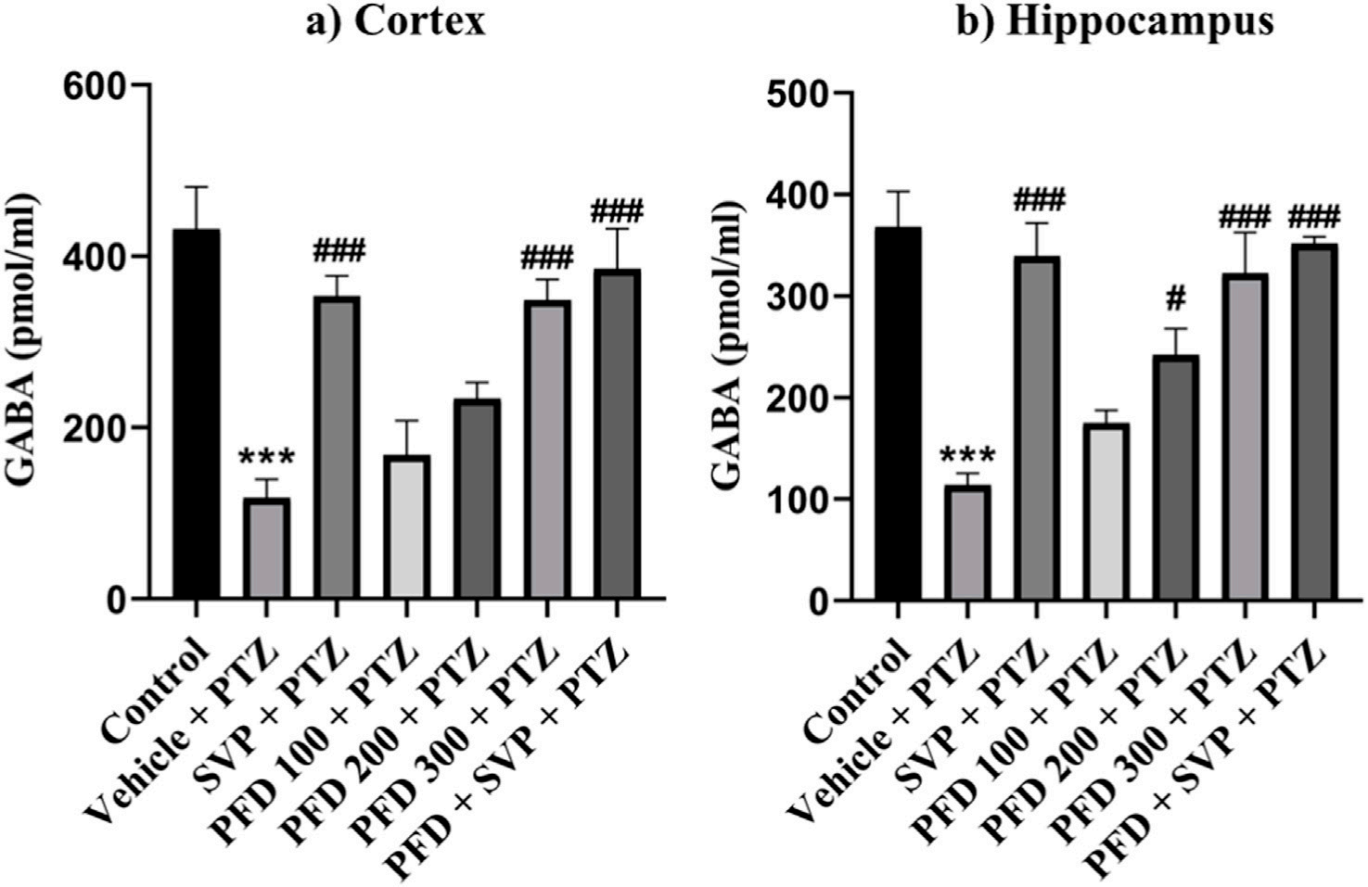
Effect PFD on Levels of GABA in the Hippocampus and Cortex of the PTZ-induced kindled mice. Data were analyzed by one-way ANOVA followed by Tukey’s multiple comparison tests (*n* = 6) and expressed as mean ± SEM, **(A)** GABA levels in Cortex F (6,35) = 12.1; ***p < 0.001 Control vs. PTZ + vehicle, ###p < 0.001 PTZ + vehicle vs. SVP + PTZ, PFD 300 + PTZ, PFD300 + SVP Q20 + PTZ, and **(B)** GABA levels in Hippocampus F (6,35) = 13.9, ***p < 0.001 Control vs. PTZ + vehicle, #p < 0.05 PTZ + vehicle vs. SVP + PTZ, ###p < 0.001 PTZ + vehicle vs. SVP + PTZ, PFD 300 + PTZ, PFD300 + SVP Q20 + PTZ.

**FIGURE 11:**
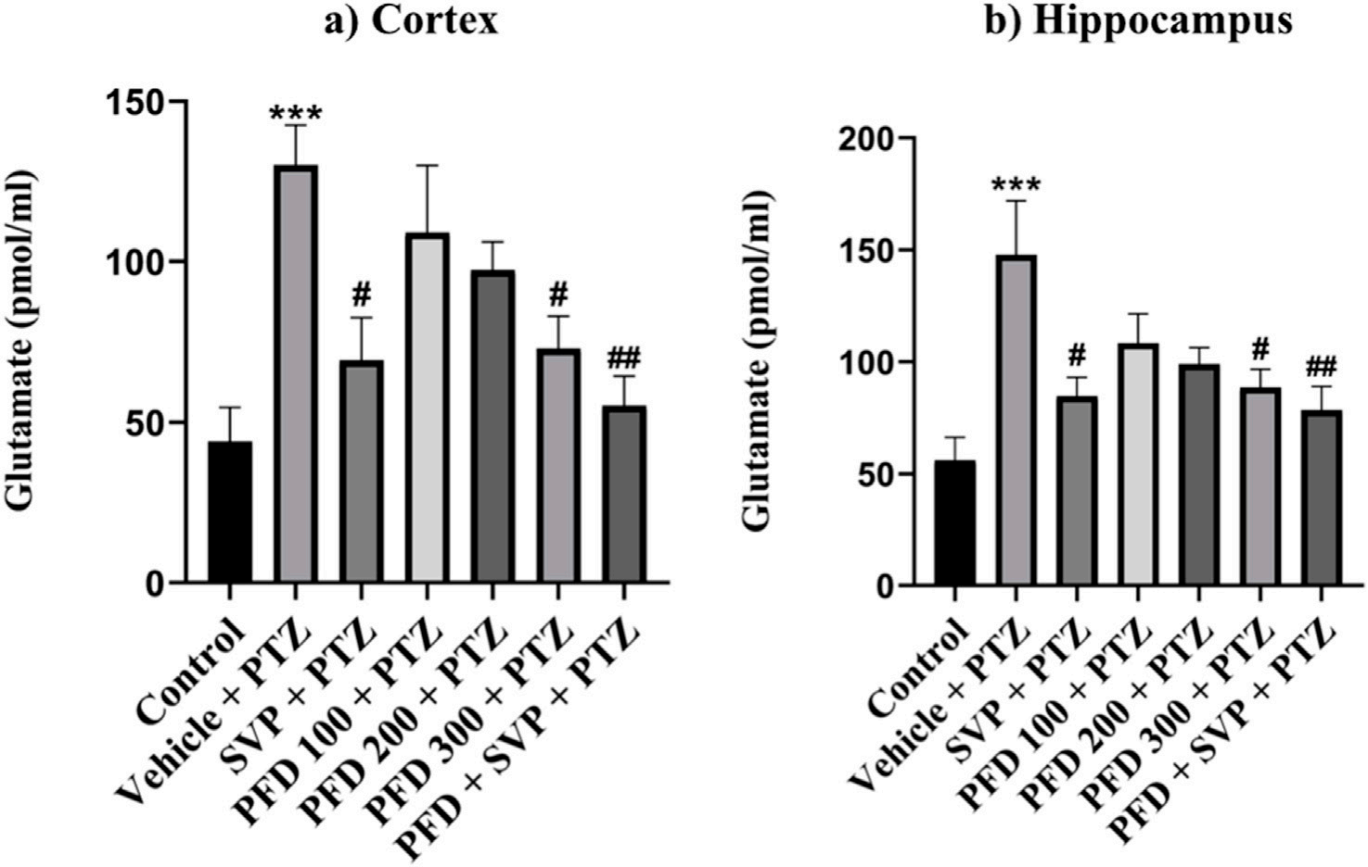
Effect PFD on Levels of Glutamate in the Hippocampus and Cortex of the PTZ-induced kindled mice. Data were analyzed by one-way ANOVA followed by Tukey’s multiple comparison tests (*n* = 6) and expressed as mean ± SEM, **(A)** Glutamate levels in Cortex F (6,35) = 5.83; ***p < 0.001 Control vs. PTZ + vehicle, #p < 0.05 PTZ + vehicle vs. SVP + PTZ, PFD 300 + PTZ, ##p < 0.01 PTZ + vehicle vs. PFD300 + SVP + PTZ and **(B)** Glutamate levels in Hippocampus F (6,35) = 4.93 ***p < 0.001 Control vs. PTZ + vehicle, #p < 0.05 PTZ + vehicle vs. SVP + PTZ, PFD 300 + PTZ, ##p < 0.01 PTZ + vehicle vs. PFD300 + SVP + PTZ.

The levels of GABA were significantly reduced in the Vehicle + PTZ group (p < 0.001) compared to the control group. However, the GABA concentration in the hippocampus significantly increased in the PFD 200 mg/kg *p.o* + PTZ (p < 0.05) and PFD 300 mg/kg + PTZ (p < 0.001) groups compared to the Vehicle + PTZ group. In the cortex, the PFD 300 mg/kg *p.o* + PTZ group (p < 0.001) also exhibited a significant increase in GABA levels compared to the Vehicle + PTZ group. Both the SVP + PTZ and PFD 300 mg/kg + SVP + PTZ groups showed a significant increase in GABA levels (p < 0.001) in the hippocampus and cortex of kindled mice compared to the Vehicle + PTZ group. Furthermore, glutamate levels in the hippocampus and cortex were significantly increased in the Vehicle + PTZ group (p < 0.001) compared to the control group. However, the PFD 300 mg/kg + PTZ group demonstrated a significant decrease in glutamate levels (p < 0.05) in both the hippocampus and cortex compared to the Vehicle + PTZ group. Additionally, the SVP + PTZ (p < 0.05) and PFD 300 mg/kg + SVP + PTZ (p < 0.01) groups also showed significant decreases in glutamate levels compared to the Vehicle + PTZ group (Figures 10, 11).

### 4.10 Effects of pirfenidone administration on liver enzyme levels in pentylenetetrazol-induced kindling in mice

**FIGURE 12:**
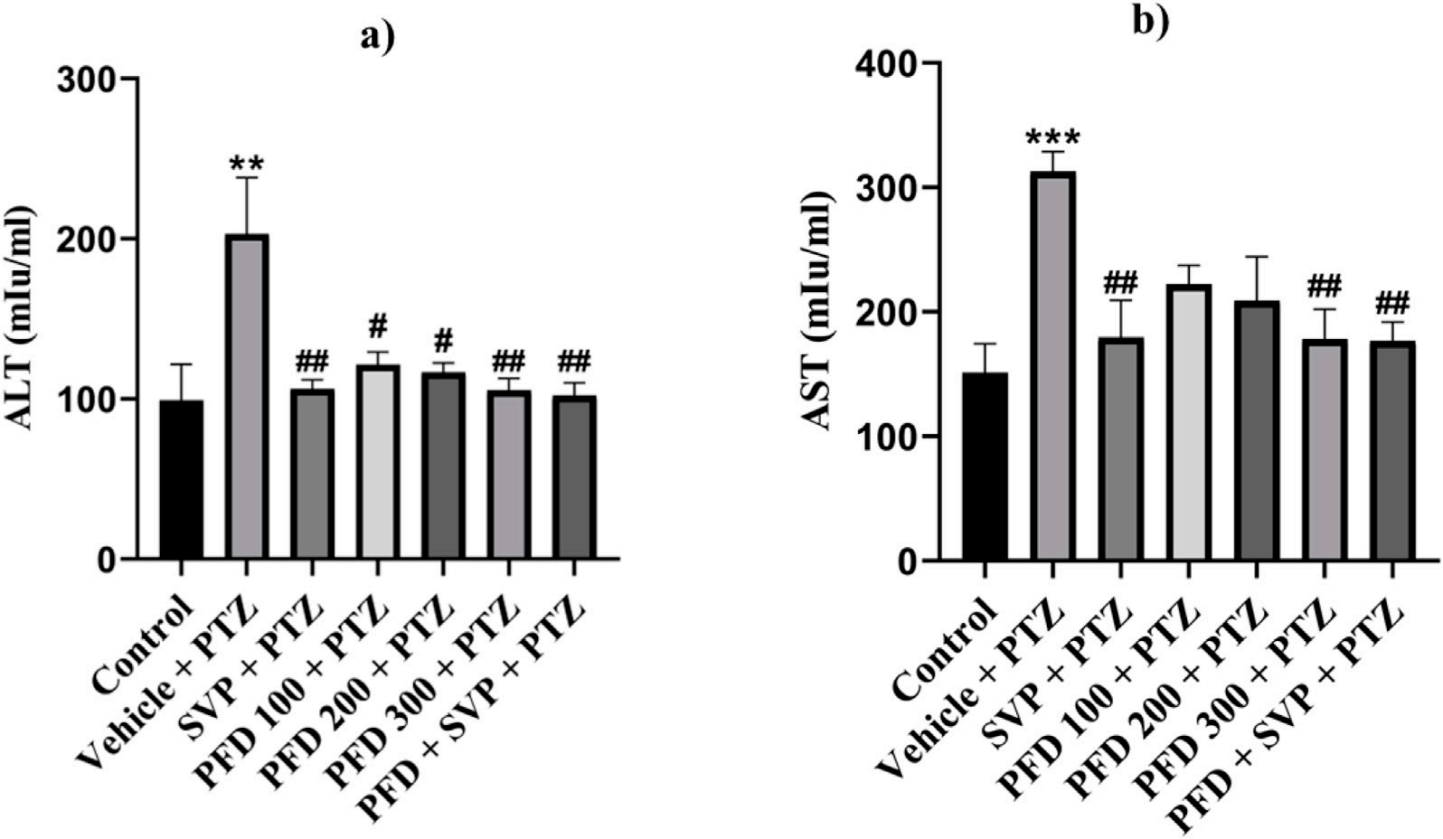
The effect of Pirfenidone administration on levels of ALT and AST enzymes in PTZ-induced kindled mice. Data were analyzed by one-way ANOVA followed by Tukey’s multiple comparison test (*n* = 6) and expressed as mean ± SEM. Results were considered significant with p-value ≤ 0.05. **(A)** presents ALT levels, F (6,35) = 4.70 **p < 0.01 Control vs. PTZ + vehicle; #p < 0.05 PTZ + vehicle vs. PFD 100 + PTZ, PFD 200 + PTZ; ##p < 0.01 PTZ + vehicle vs. SVP + PTZ, PFD 300 + PTZ, PFD 300 + SVP + PTZ. **(B)** presents AST levels, F (6,35) = 5.05 ***p < 0.001 Control vs. PTZ + vehicle; ##p < 0.01 PTZ + vehicle vs. SVP + PTZ; PFD300 + PTZ; PFD300 + SVP + PTZ.

The levels of AST (p < 0.001) and ALT (p < 0.01) enzymes were significantly increased in the Vehicle + PTZ group in comparison to the control group. On the other hand, administration of SVP + PTZ (p < 0.01), PFD 300 + PTZ (p < 0.01), and SVP + PFD 300 + PTZ (p < 0.01) significantly decreased the level of AST in kindled mice. On the other hand, SVP + PTZ (p < 0.01), PFD 100 + PTZ (p < 0.05), PFD 200 + PTZ (p < 0.05), PFD 300 + PTZ (p < 0.01) and SVP + PFD 300 + PTZ (p < 0.01) groups significantly decreased the levels of the ALT of kindled mice in comparison to the vehicle + PTZ group (Figure 12).

### 4.11 Effects of pirfenidone histopathological analysis of pentylenetetrazol-induced kindling in mice

**FIGURE 13:**
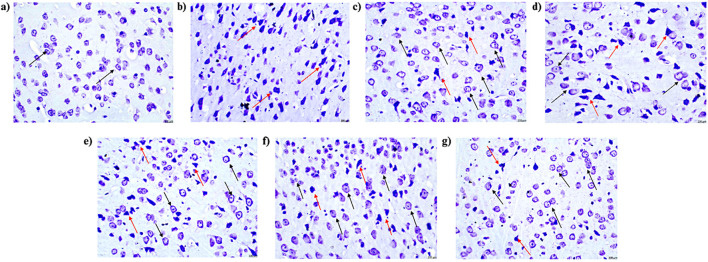
Microscopic examination of the cortical tissue samples using ×400 magnification using cresyl violet dye indicating neuronal damage (n = 3) **(A)** Control **(B)** PTZ + Vehicle **(C)** SVP + Vehicle **(D)** PFD 100 + PTZ **(E)** PFD 200 + PTZ **(F)** PFD 300 + PTZ **(G)** PFD 300 + SVP + PTZ. **(A)** Control: Black arrows indicate a normal histological appearance of the CA1 region of the cortex, with no signs of pyknosis, cellular disintegration, vacuolations, or apoptotic changes observed. **(B)** Vehicle + PTZ: The section shows a severely damaged CA1 region of the cortex with extensive cellular disintegration, marked pyknosis, and degenerative changes (red arrow), indicating significant neuronal damage. **(C)** SVP (200 mg/kg) + PTZ: The section reveals a minimally damaged CA1 region of the cortex with minimal cellular disintegration, mild pyknosis, and degenerative changes (red arrow). Numerous healthy neurons (black arrow) are still visible, indicating partial neuroprotection by SVP. **(D)** PFD (100 mg/kg) + PTZ: The section displays a moderately damaged CA1 region of the cortex with moderate cellular disintegration, marked pyknosis, and degenerative changes (red arrow). However, numerous healthy neurons (black arrow) are also present, indicating limited neuroprotection at this dose. **(E)** PFD (200 mg/kg) + PTZ: The section shows mildly damaged CA1 region of the cortex, similar to the 100 mg/kg dose, with moderate cellular disintegration, marked pyknosis, and degenerative changes (red arrow). Numerous healthy neurons (black arrow) are still visible. **(F)** PFD (300 mg/kg) + PTZ: The section shows minimally damaged CA1 region of the cortex, with mild cellular disintegration, pyknosis, and degenerative changes (red arrow). Numerous healthy neurons (black arrow) are present, suggesting significant neuroprotection at this higher dose. **(G)** PFD (300 mg/kg) + SVP (200 mg/kg) + PTZ: The section shows a minimally damaged CA1 region of the cortex, with minimal cellular disintegration, mild pyknosis, and degenerative changes (red arrow). Numerous healthy neurons (black arrow) are observed, indicating a synergistic neuroprotective effect when PFD (300 mg/kg) is combined with SVP (200 mg/kg).

**FIGURE 14:**
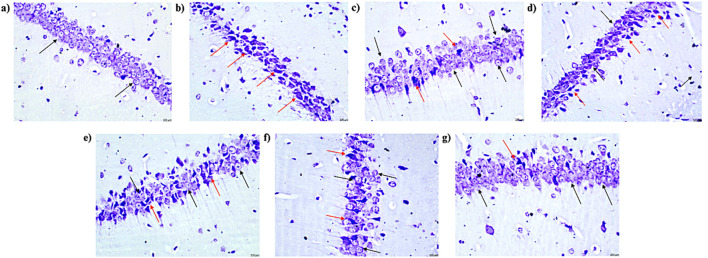
Microscopic examination of the hippocampal tissue samples using ×400 magnification using cresyl violet dye indicating neuronal damage (n = 3) **(A)** Control **(B)** PTZ + Vehicle **(C)** SVP + Vehicle **(D)** PFD 100 + PTZ **(E)** PFD 200 + PTZ **(F)** PFD 300 + PTZ **(G)** PFD 300 + SVP + PTZ. **(A)** Control: Black arrows indicate a normal histological appearance of the CA1 region of the hippocampus, with no signs of pyknosis, cellular disintegration, vacuolations, or apoptotic changes observed. **(B)** Vehicle + PTZ: The section shows a severely damaged CA1 region of the hippocampus with extensive cellular disintegration, marked pyknosis, and degenerative changes (red arrow), indicating significant neuronal damage. **(C)** SVP (200 mg/kg) + PTZ: The section reveals a minimally damaged CA1 region of the hippocampus with minimal cellular disintegration, mild pyknosis, and degenerative changes (red arrow). Numerous healthy neurons (black arrow) are still visible, indicating partial neuroprotection by SVP. **(D)** PFD (100 mg/kg) + PTZ: The section displays a moderately damaged CA1 region of the hippocampus with moderate cellular disintegration, marked pyknosis, and degenerative changes (red arrow). However, numerous healthy neurons (black arrow) are also present, indicating limited neuroprotection at this dose. **(E)** PFD (200 mg/kg) + PTZ: The section shows a mildly damaged CA1 region of the hippocampus, similar to the 100 mg/kg dose, with moderate cellular disintegration, marked pyknosis, and degenerative changes (red arrow). Numerous healthy neurons (black arrow) are still visible. **(F)** PFD (300 mg/kg) + PTZ: The section shows minimally damaged CA1 region of the hippocampus, with mild cellular disintegration, pyknosis, and degenerative changes (red arrow). Numerous healthy neurons (black arrow) are present, suggesting significant neuroprotection at this higher dose. **(G)** PFD (300 mg/kg) + SVP (200 mg/kg) + PTZ: The section shows a minimally damaged CA1 region of the hippocampus, with minimal cellular disintegration, mild pyknosis, and degenerative changes (red arrow). Numerous healthy neurons (black arrow) are observed, indicating a synergistic neuroprotective effect when PFD (300 mg/kg) is combined with SVP (200 mg/kg).

The control group displayed normal histological features in the CA1 region of the hippocampus and cortex, with no signs of pyknosis, cellular disintegration, vacuolations, or apoptotic changes, as observed with CV staining at ×400 magnification. In contrast, the vehicle + PTZ group exhibited severe damage in the CA1 region and cortex, characterized by significant cellular disintegration, marked pyknosis, and degenerative changes (red arrow). The PFD 100 + PTZ and PFD 200 + PTZ groups showed moderate damage, with visible cellular disintegration and pyknosis, though numerous healthy neurons (black arrow) were present. In the PFD 300 + PTZ and SVP + PTZ groups, damage was mild, with reduced cellular disintegration and pyknosis. The PFD 300 + SVP + PTZ group demonstrated minimal damage, indicating the protective effects of the combined treatment (Figures 13, 14).

## 5 Discussion

The study investigated the effects of PFD on seizure activity, cognition, oxidative stress, inflammatory markers, neurotransmitter levels, and liver enzyme levels, in a PTZ-induced kindling model of epilepsy. It was observed that higher doses of PFD (200 mg/kg and 300 mg/kg) increased the seizure threshold current (STC) significantly during the ICES test, indicating a protective effect against seizures. This protection may be attributed to findings from an *in vivo* study, where a single dose of PFD (325 mg/kg) administered 90 min after KA (kainic acid) injection significantly reduced neuronal loss specifically in the CA1 and CA3 areas of the hippocampus. These results suggest that PFD may protect neurons from excitotoxic damage associated with seizures (Castro-Torres et al., 2014). Seizures often lead to mitochondrial dysfunction, which exacerbates neuronal damage and lowers seizure thresholds. PFD may protect mitochondrial function by preventing oxidative damage, thus preserving cellular energy balance and contributing to an increased STC (Castro-Torres et al., 2015; Bozkurt et al., 2022).

The PTZ-induced kindling model is extensively used to investigate the mechanisms of epilepsy and to evaluate the efficacy of anticonvulsant drugs as it is favored for exploring long-term neurochemical and structural changes in the brain (Khatoon et al., 2021). Repetitive administration of a sub-convulsive dose of PTZ (25 mg/kg, *i.p.*) led to chemical kindling, resulting in the emergence of seizures resembling generalized tonic-clonic seizures (Aleshin et al., 2023). SVP is among the most widely used antiepileptic drugs and is proven to show anticonvulsant effects in the PTZ model (Wang et al., 2020). In this study, PFD administration reduced seizure severity significantly compared to the PTZ + vehicle group. The exact mechanisms behind this increase in STC and reduction in seizure severity may include the reduction of inflammation and oxidative stress in the brain, which are key contributors to seizure activity. PFD may induce protection against seizures by increasing the brain’s resistance to seizure-inducing stimuli, thereby making it more difficult for seizures to be triggered (Bozkurt et al., 2022).

Oxidative stress, characterized by increased reactive oxygen species (ROS) and lipid peroxidation, contributes significantly to neuronal damage in epilepsy (Madireddy and Madireddy, 2023). Elevated malondialdehyde (MDA) levels and reduced antioxidant enzyme activity (SOD, catalase, and GSH) in the Vehicle + PTZ group indicate increased oxidative stress. PFD treatment, particularly at 300 mg/kg, effectively restored antioxidant profiles, reducing MDA levels and enhancing antioxidant enzyme activities. These findings are supported by studies demonstrating PFD’s antioxidant properties in mitigating oxidative damage in various models of neuroinflammation and tissue injury (Salazar-Montes et al., 2008; Dutta et al., 2017; Aimo et al., 2022; Bozkurt et al., 2022). By enhancing antioxidant defenses, PFD may prevent ROS-induced excitotoxicity and protect against mitochondrial dysfunction, thereby preserving neuronal function (Dutta et al., 2017).

Acetylcholinesterase (AChE) plays a critical role in regulating cholinergic transmission, which is essential for neuronal excitability and cognitive function (Komali et al., 2021). Elevated AChE activity, often observed in epilepsy, disrupts cholinergic signaling by accelerating acetylcholine hydrolysis, leading to increased neuronal excitability and cognitive impairments such as memory and learning deficits (Alyami et al., 2022). In this study, PFD significantly reduced AChE activity in the hippocampus and cortex, thereby preserving acetylcholine levels and improving cognitive outcomes, as evidenced by enhanced performance in behavioral tasks like passive avoidance and elevated plus maze tests. Additionally, PFD’s antioxidant properties may contribute to this effect by mitigating oxidative stress, a known contributor to cholinergic dysfunction in epilepsy (Evani et al., 2020). Oxidative stress can impair cholinergic neurons, further increasing AChE activity and aggravating cognitive deficits (Madireddy and Madireddy, 2023). Previous studies have shown that reducing AChE activity not only improves cognitive performance but also stabilizes neuronal excitability, thereby offering neuroprotective effects in epilepsy (Komali et al., 2021; Alyami et al., 2022).

Epilepsy is often accompanied by cognitive deficits, primarily due to hippocampal dysfunction and neuroinflammation (Novak et al., 2022). Behavioral tests such as transfer latency in the elevated plus maze and step-down latency in the passive avoidance test demonstrated that PFD, particularly at 300 mg/kg, significantly improved both acquisition and retention latency. This suggests enhanced memory retention and preserves cognitive function. Such improvements align with reductions in pro-inflammatory cytokines (IL-6, TNF-α, IL-1β, and HMGB1) and oxidative markers observed in this study.

Neuroinflammation disrupts hippocampal plasticity, impairing spatial learning and memory (Suleymanova, 2021). PFD’s ability to reduce cytokines like HMGB1 and TLR-4 likely stabilized the neuronal microenvironment, supporting hippocampal function. Furthermore, the restoration of GABA and reduction of glutamate levels in the hippocampus and cortex may have alleviated cognitive impairments, consistent with prior research linking GABAergic dysfunction and glutamate excitotoxicity to cognitive decline in epilepsy on mice model of cognitive dysfunction (Zhu et al., 2020; Zhou et al., 2022).

PFD administration significantly reduced pro-inflammatory cytokines such as IL-1β, IL-6, and TNF-α, in the hippocampus and cortex, likely due to its ability to inhibit the release of these pro-inflammatory cytokines, which are known to enhance neuronal excitability and promote seizures, while simultaneously enhancing the production of anti-inflammatory cytokines like IL-10 (Soltani Khaboushan et al., 2022). Although data on PFD treatment for neurological disorders is limited, there is evidence from other studies that PFD has demonstrated protective effects against silica-induced lung injury, where it reduced the release of pro-inflammatory cytokines such as TNF-α, IL-1β, and IL-6 in mice (Cao et al., 2022) Additionally, PFD reduced levels of IL-1β and IL-18 in animal models of pulmonary hypertension by suppressing the NOD-like pyrin domain-containing protein 3 (NLRP3) inflammasome (Brunet et al., 2022). This mechanism is especially pertinent to neurological disorders, where the activation of the NLRP3 inflammasome, along with pro-IL-1β, can trigger TLR4 activation. This, in turn, initiates NF-κB signaling, which drives the transcription of NF-κB-dependent genes in the nucleus evident in epilepsy animal models (Liu et al., 2017; Sharawy and Serrya, 2020; Cai and Lin, 2022).

In recent studies, PFD has been identified as a significant modulator of the HMGB1/TLR4 signaling axis, which is crucial in regulating neuroinflammation and neuronal plasticity (Zhao et al., 2014). In murine models of acute pancreatitis, PFD treatment resulted in reduced serum levels of HMGB1 and C-reactive protein (Palathingal Bava et al., 2022), two inflammatory markers that have been linked to poor outcomes in clinical studies of epilepsy (Zhong et al., 2019; Chen et al., 2023a). Similarly, in this study, the PFD administration effectively suppressed the levels of HMGB1 and TLR-4 in the hippocampus and cortex in a dose-dependent manner in comparison to the control group. This suppression is particularly important because HMGB1, when released extracellularly, binds to TLR4 and activates downstream pro-inflammatory pathways, including MAPK. These pathways subsequently lead to the increased production of growth factors such as TGF-β, which are known to exacerbate neuroinflammatory responses, disrupt the BBB, and contribute to the onset of seizures (Zhang et al., 2017). The ability of PFD to modulate TGF-β and other cytokines involved in inflammation, as observed in this study, suggests that it can stabilize the neuronal environment by reducing excitatory conditions that might lead to seizures. By inhibiting the activity of TGF-β and reducing its levels, PFD potentially diminishes the inflammatory response, thereby mitigating the harmful effects associated with epilepsy. This effect could be attributed to the reduction of HMGB1/TLR4 signaling, which dampens the inflammatory response and leads to a lower production of TGF-β (Zhang et al., 2017). Simultaneously, the HMGB1/TLR4 axis can influence the Smad signaling pathway, which is required for the downstream effects of TGF-β. Inhibiting HMGB1/TLR4 signaling may reduce the phosphorylation and activation of Smad2/3, thereby decreasing TGF-β signaling and its associated effects. This inhibition may help to stabilize the neuronal environment, reduce the likelihood of seizures, and protect against seizure-induced neuronal damage (Song et al., 2021; Chen et al., 2023b).

The GABAergic system, responsible for inhibitory neurotransmission, is also adversely affected by the activation of TLR4 signaling. Activation of TLR4 by HMGB1 leads to a decrease in GABA synthesis and postsynaptic GABA receptor activity, contributing to the imbalance between excitation and inhibition in the brain. This imbalance promotes neuroinflammation and neurodegeneration, both of which are critical factors in the development and exacerbation of epilepsy (Yan et al., 2015). Elevated extracellular glutamate levels can lead to hyperexcitability of neurons, which is a key factor in the initiation and propagation of seizures. Studies have shown that during seizures, there is often a significant increase in extracellular glutamate concentrations, which can reach neurotoxic levels, further exacerbating the excitatory environment in the brain (During and Spencer, 1993). A reduction in GABA levels can lower the threshold for seizure generation, as the inhibitory control over neuronal firing is diminished. This decrease in GABAergic inhibition is a critical factor in seizure development, especially when combined with elevated glutamate levels (Ueda et al., 2001). It was observed that PFD administration significantly increased GABA levels and decreased glutamate levels compared to the control group. Additionally, under pathological conditions, excessive glutamate release and impaired clearance can lead to excitotoxicity, a key contributor to neuronal damage and seizure activity. HMGB1 release in response to glutamate-induced excitotoxicity further activates TLR4, amplifying the inflammatory response and neuronal damage, creating a vicious cycle of neuroinflammation and excitotoxicity. PFD’s role in reducing HMGB1 and TLR4 levels may interrupt this cycle, reducing glutamate levels and protecting neurons from excitotoxic damage. This reduction in extracellular glutamate, coupled with the preservation of GABA levels, is crucial for lowering the threshold for seizure generation and maintaining neuronal stability. The ability of PFD to reduce HMGB1 and TLR4 levels could help preserve GABAergic transmission, thereby restoring the balance between excitatory and inhibitory neurotransmission, which is essential for controlling seizure activity (Zou and Crews, 2015).

Certain AEDs are known to cause hepatotoxicity, which can range from mild, transient elevations in liver enzymes to severe liver injury leading to acute liver failure. Among these, phenytoin, SVP, and carbamazepine are particularly associated with liver toxicity. These ASMs undergo extensive hepatic metabolism, which can result in the formation of toxic metabolites that contribute to liver injury (Vidaurre et al., 2017). In this study, it was observed that PFD effectively reduced elevated AST and ALT levels, suggesting a protective effect on liver function. In an investigation into the effects of PFD on D-galactosamine and lipopolysaccharide-induced acute hepatotoxicity in rats, PFD significantly reduced the elevated ALT and AST levels (Wang et al., 2008). Similarly, in a study on liver fibrosis induced by carbon tetrachloride (CCl4) in mice, PFD treatment resulted in a marked decrease in ALT and AST levels, highlighting its effectiveness in mitigating liver damage associated with fibrosis (Xiao et al., 2016). Further supporting these findings, a study on experimental liver cirrhosis demonstrated that PFD treatment significantly reduced ALT and AST levels in cirrhotic rats, along with a decrease in other markers of liver damage (García et al., 2002). Additionally, in patients with chronic hepatitis C, long-term treatment with PFD was associated with a trend toward normalization of ALT and AST levels and improvements in other liver function indicators (Flores-Contreras et al., 2014).

Histopathological analysis confirmed PFD’s neuroprotective effects. In the vehicle + PTZ group, severe neuronal damage was observed in both the hippocampus and cortex, characterized by pronounced pyknosis, cellular disintegration, and degenerative changes. Severe neuronal damage, including pyknosis and cellular disintegration, observed in the Vehicle + PTZ group was significantly alleviated in PFD-treated groups, particularly at 300 mg/kg. These findings are consistent with studies demonstrating PFD’s ability to preserve neuronal architecture in models of neuroinflammation and excitotoxicity (Dutta et al., 2017; Bozkurt et al., 2022). PFD treatment, particularly at the 300 mg/kg dose, significantly alleviated these histopathological abnormalities. The hippocampus and cortex of PFD-treated animals exhibited a reduction in pyknosis and cellular disintegration, with more preserved neuronal architecture compared to the vehicle + PTZ group. This protective effect was most pronounced in the PFD 300 mg/kg + SVP combination group, which showed minimal neuronal damage, suggesting a potential synergistic effect between PFD and SVP. The preservation of hippocampal and cortical neurons likely contributed to improved cognitive outcomes and reduced seizure severity. Additionally, the combination of PFD with SVP showed minimized neuronal damage, indicating potential combinatory benefits in epilepsy treatment. The histopathological findings corroborate the biochemical and behavioral results of this study. Additionally, by reducing pro-inflammatory cytokines and modulating antioxidant enzyme activity, PFD likely stabilizes the neuronal microenvironment, preserving structural integrity in the hippocampus and cortex.

Due to these properties, PFD has also shown possible benefits during the secondary phase of Traumatic brain injury, where neuroinflammation causes worsening neurological outcomes. It was observed that PFD reduced neuroinflammation by decreasing levels of neuron-specific enolase (NSE) and S100B (biomarkers of brain injury) and improved neurological outcomes in the Garcia test (Bozkurt et al., 2022). Similarly, administration of PFD in patients with secondary progressive multiple sclerosis (SPMS) has been shown to stabilize or even improve neurological functions over up to 2 years. Patients treated with PFD had fewer relapses and showed improvement in bladder function, another common symptom of epilepsy where loss of bladder control is observed (Walker et al., 2005). This suggests that PFD may reduce cognitive and physical dysfunction and slow the progression of neurological disabilities by mitigating inflammation and oxidative stress (Walker et al., 2005). Relatively, similar results where PFD’s anti-inflammatory activity has been assessed in various organ systems, including the heart (Aimo et al., 2022) kidneys (Bai et al., 2021) liver (Escutia-Gutiérrez et al., 2021) and lungs (Ruwanpura et al., 2020).

While the present study demonstrates the anticonvulsant, neuroprotective, and cognitive-enhancing effects of pirfenidone (PFD) in a PTZ-induced kindling model of epilepsy, it is important to acknowledge the limitation of not evaluating the long-term effects of PFD treatment. Chronic epilepsy management requires sustained efficacy and safety over extended durations, particularly concerning cognitive outcomes and seizure control. The current findings provide critical insights into the acute and sub-chronic effects of PFD; however, further investigations are necessary to determine its potential as a chronic therapeutic agent.

This study provides evidence of PFD anticonvulsant and neuroprotective effects in the PTZ-induced kindling model of epilepsy The 6-week treatment period was sufficient to demonstrate significant improvements in seizure control, cognitive performance, and biochemical modulation, establishing a strong foundation for PFD’s therapeutic potential. The study also observed the modulation of the HMGB1/TLR4 pathway as a key mechanism of PFD’s effects, supported by reductions in pro-inflammatory markers. Additionally, the evaluation of three doses (100, 200, and 300 mg/kg) demonstrated that 300 mg/kg was the most effective, suggesting a strong dose-response relationship.

However, this study did not explore the long-term effects of PFD, which are essential for chronic conditions like epilepsy. Investigating its sustained efficacy, safety, and cognitive benefits over extended durations remains a vital next step. Future studies should focus on assessing the long-term impact of PFD through chronic treatment regimens in animal models, incorporating neurobehavioral assessments, biochemical analyses, and histopathological evaluations over extended timeframes. Additionally, studies exploring the molecular mechanisms underlying the sustained modulation of neuroinflammation, oxidative stress, and neurotransmitter balance by PFD would provide a deeper understanding of its therapeutic potential. Simultaneously, expanding the dose range and use of placebo in future studies could further refine the therapeutic index and optimize clinical translation. Lastly, while male mice were used to reduce variability and focus on establishing baseline efficacy, future studies should include both sexes to evaluate potential gender-specific responses to PFD.

## 6 Conclusion

PFD exhibited significant anti-inflammatory, anticonvulsant, and neuroprotective effects in a PTZ-induced kindling model of epilepsy. It effectively reduced seizure severity, enhanced cognitive performance, and modulated oxidative stress by decreasing malondialdehyde (MDA) levels and boosting antioxidant enzymes (SOD, catalase, GSH). Additionally, PFD’s ability to reduce pro-inflammatory cytokines and modulate neurotransmitter levels suggests a protective role in mitigating neuroinflammation and excitotoxicity, both of which are critical in the pathogenesis of epilepsy. Furthermore, PFD displayed hepatoprotective properties by reducing elevated liver enzyme levels, indicating its potential safety in long-term use. Histopathological analysis confirmed its neuroprotective role, preserving hippocampal and cortical integrity. These findings indicate that PFD holds potential as an adjunct therapy for epilepsy. However, additional research is necessary to investigate its long-term effectiveness, safety, and applicability across various epilepsy models and its potential for clinical use.

## Data Availability

The original contributions presented in the study are included in the article/supplementary material, further inquiries can be directed to the corresponding author.

## References

[B1] Agarwal N. B. Jain S. Nagpal D. Agarwal N. K. Mediratta P. K. Sharma K. K. (2013). Liposomal formulation of curcumin attenuates seizures in different experimental models of epilepsy in mice. Fundam. Clin. Pharmacol. 27, 169–172. 10.1111/j.1472-8206.2011.01002.x 22044441

[B2] Aimo A. Spitaleri G. Panichella G. Lupón J. Emdin M. Bayes-Genis A. (2022). Pirfenidone as a novel cardiac protective treatment. Heart Fail Rev. 27, 525–532. 10.1007/s10741-021-10175-w 34671871 PMC8898227

[B3] Akyüz E. Doğanyiğit Z. Paudel Y. N. Kaymak E. Yilmaz S. Uner A. (2020). Increased ACh-associated immunoreactivity in autonomic centers in PTZ kindling model of epilepsy. Biomedicines 8, 113. 10.3390/biomedicines8050113 32397136 PMC7277646

[B4] Alam Md. N. Singh L. Khan N. A. Asiri Y. I. Hassan M. Z. Afzal O. (2023). Ameliorative effect of ethanolic extract of moringa oleifera leaves in combination with curcumin against PTZ-induced kindled epilepsy in rats: *in vivo* and *in silico* . Pharmaceuticals 16, 1223. 10.3390/ph16091223 37765031 PMC10534968

[B5] Aleshin V. A. Graf A. V. Artiukhov A. V. Ksenofontov A. L. Zavileyskiy L. G. Maslova M. V. (2023). Pentylenetetrazole-induced seizures are increased after kindling, exhibiting vitamin-responsive correlations to the post-seizures behavior, amino acids metabolism and key metabolic regulators in the rat brain. Int. J. Mol. Sci. 24, 12405. 10.3390/ijms241512405 37569781 PMC10418815

[B6] Alyami N. M. Abdi S. Alyami H. M. Almeer R. (2022). Proanthocyanidins alleviate pentylenetetrazole-induced epileptic seizures in mice via the antioxidant activity. Neurochem. Res. 47, 3012–3023. 10.1007/s11064-022-03647-4 35838827

[B7] Babovic-Vuksanovic D. Petrovic L. Knudsen B. E. Plummer T. B. Parisi J. E. Babovic S. (2004). Survival of human neurofibroma in immunodeficient mice and initial results of therapy with pirfenidone. Biomed. Res. Int. 2004, 79–85. 10.1155/S1110724304308107 PMC548804 15240917

[B8] Bai X. Nie P. Lou Y. Zhu Y. Jiang S. Li B. (2021). Pirfenidone is a renal protective drug: mechanisms, signalling pathways, and preclinical evidence. Eur. J. Pharmacol. 911, 174503. 10.1016/j.ejphar.2021.174503 34547247

[B9] Bozkurt I. Ozturk Y. Guney G. Arslan B. Gulbahar O. Guvenc Y. (2022). Effects of pirfenidone on experimental head injury in rats. Int. J. Clin. Exp. Pathol. 15, 20–28. 35145580 PMC8822207

[B10] Brunet D. Van S. D. Masson B. Antigny F. Haddad F. Kloeckner M. (2022). Left ventricular diastolic dysfunction in chronic thromboembolic pulmonary hypertension. J. Heart Lung Transplant. 41, S26–S27. 10.1016/j.healun.2022.01.057

[B11] Cai M. Lin W. (2022). The function of NF-kappa B during epilepsy, a potential therapeutic target. Front. Neurosci. 16, 851394. 10.3389/fnins.2022.851394 35360161 PMC8961383

[B12] Cao Z. Liu Y. Zhang Z. Yang P. Li Z. Song M. (2022). Pirfenidone ameliorates silica-induced lung inflammation and fibrosis in mice by inhibiting the secretion of interleukin-17A. Acta Pharmacol. Sin. 43, 908–918. 10.1038/s41401-021-00706-4 34316030 PMC8976043

[B13] Castro-Torres R. D. Chaparro-Huerta V. Flores-Soto M. E. Bañuelos-Pineda J. Camins A. Orozco-Suárez S. A. (2014). A single dose of pirfenidone attenuates neuronal loss and reduces lipid peroxidation after kainic acid-induced excitotoxicity in the pubescent rat Hippocampus. J. Mol. Neurosci. 52, 193–201. 10.1007/s12031-013-0121-6 24142572

[B14] Castro-Torres R. D. Chaparro-Huerta V. Flores-Soto M. E. Jave-Suárez L. Camins A. Armendáriz-Borunda J. (2015). Pirfenidone attenuates microglial reactivity and reduces inducible nitric oxide synthase mRNA expression after kainic acid-mediated excitotoxicity in pubescent rat Hippocampus. J. Mol. Neurosci. 56, 245–254. 10.1007/s12031-015-0509-6 25854776

[B15] Chen Y. Chen X. Liang Y. (2023a). Meta-analysis of HMGB1 levels in the cerebrospinal fluid and serum of patients with epilepsy. Neurol. Sci. 44, 2329–2337. 10.1007/s10072-023-06720-0 36933099

[B16] Chen Y. Nagib M. M. Yasmen N. Sluter M. N. Littlejohn T. L. Yu Y. (2023b). Neuroinflammatory mediators in acquired epilepsy: an update. Inflamm. Res. 72, 683–701. 10.1007/s00011-023-01700-8 36745211 PMC10262518

[B17] During M. J. Spencer D. D. (1993). Extracellular hippocampal glutamate and spontaneous seizure in the conscious human brain. Lancet 341, 1607–1610. 10.1016/0140-6736(93)90754-5 8099987

[B18] Dutta D. Kumar S. L. H. Grewal A. K. (2017). Neuroprotective effect of pirfenidone on scopolamine induced cognitive impairment and oxidative stress. Indian J. Physiology Pharmacol. 61, 416–429.

[B19] Ellman G. L. Courtney K. D. Andres V. Featherstone R. M. (1961). A new and rapid colorimetric determination of acetylcholinesterase activity. Biochem. Pharmacol. 7, 88–95. 10.1016/0006-2952(61)90145-9 13726518

[B20] Escutia-Gutiérrez R. Rodríguez-Sanabria J. S. Monraz-Méndez C. A. García-Bañuelos J. Santos-García A. Sandoval-Rodríguez A. (2021). Pirfenidone modifies hepatic miRNAs expression in a model of MAFLD/NASH. Sci. Rep. 11, 11709. 10.1038/s41598-021-91187-2 34083664 PMC8175718

[B21] Evani S. J. Karna S. L. R. Seshu J. Leung K. P. (2020). Pirfenidone regulates LPS mediated activation of neutrophils. Sci. Rep. 10, 19936. 10.1038/s41598-020-76271-3 33203891 PMC7672086

[B22] Flores-Contreras L. Sandoval-Rodríguez A. S. Mena-Enriquez M. G. Lucano-Landeros S. Arellano-Olivera I. Álvarez-Álvarez A. (2014). Treatment with pirfenidone for two years decreases fibrosis, cytokine levels and enhances CB2 gene expression in patients with chronic hepatitis C. BMC Gastroenterol. 14, 131. 10.1186/1471-230X-14-131 25064094 PMC4236537

[B23] García L. Hernández I. Sandoval A. Salazar A. Garcia J. Vera J. (2002). Pirfenidone effectively reverses experimental liver fibrosis. J. Hepatol. 37, 797–805. 10.1016/S0168-8278(02)00272-6 12445421

[B24] Ghaith A. K. El-Hajj V. G. Sanchez-Garavito J. E. Zamanian C. Ghanem M. Bon-Nieves A. (2024). Trends in the utilization of surgical modalities for the treatment of drug-resistant epilepsy: a comprehensive 10-year analysis using the national inpatient sample. Neurosurgery. 10.1227/neu.0000000000002811 38189460

[B25] Greenwald R. A. (2018). Handbook methods for oxygen radical research. Boca Raton, London, New York: CRC Press web Taylor and Francis group.

[B26] Imran M. Riaz T. Majid H. Maqsood S. (2023). Identification of botanicals using molecular biotechnology. Polym. Med. 53, 69–79. 10.17219/PIM/163119 37338286

[B27] Ivens S. Kaufer D. Flores L. P. Bechmann I. Zumsteg D. Tomkins O. (2007). TGF-beta receptor-mediated albumin uptake into astrocytes is involved in neocortical epileptogenesis. Brain 130 (Pt 2), 535–547. 10.1093/BRAIN/AWL317 17121744

[B28] Jollow D. J. Mitchell J. R. Zampaglione N. Gillette J. R. (1974). Bromobenzene-induced liver necrosis. Protective role of glutathione and evidence for 3,4-bromobenzene oxide as the hepatotoxic metabolite. Pharmacology 11, 151–169. 10.1159/000136485 4831804

[B29] Kamali A. N. Zian Z. Bautista J. M. Hamedifar H. Hossein-Khannazer N. Hosseinzadeh R. (2020). The potential role of pro-inflammatory and anti-inflammatory cytokines in epilepsy pathogenesis. Endocr. Metab. Immune Disord. Drug Targets 21, 1760–1774. 10.2174/1871530320999201116200940 33200702

[B30] Khan W. U. Salman M. Ali M. Majid H. Yar M. S. Akhtar M. (2024). Neuroprotective effects of sulforaphane in a rat model of alzheimer’s disease induced by aβ (1-42) peptides. Neurochem. Int. 179, 105839. 10.1016/J.NEUINT.2024.105839 39173832

[B31] Khatoon S. Agarwal N. B. Samim M. Alam O. (2021). Neuroprotective effect of fisetin through suppression of IL-1R/TLR Axis and apoptosis in pentylenetetrazole-induced kindling in mice. Front. Neurol. 12, 689069. 10.3389/fneur.2021.689069 34354662 PMC8333701

[B32] Khatoon S. Samim M. Dahalia M. Nidhi (2023). Fisetin provides neuroprotection in pentylenetetrazole-induced cognition impairment by upregulating CREB/BDNF. Eur. J. Pharmacol. 944, 175583. 10.1016/j.ejphar.2023.175583 36764352

[B33] Komali E. Venkataramaiah C. Rajendra W. (2021). Antiepileptic potential of Bacopa monnieri in the rat brain during PTZ-induced epilepsy with reference to cholinergic system and ATPases. J. Tradit. Complement. Med. 11, 137–143. 10.1016/j.jtcme.2020.02.011 33728274 PMC7936099

[B34] Kumar V. Sharma S. K. Nagarajan K. Dixit P. K. (2016). Effects of lycopene and sodium valproate on pentylenetetrazol-induced kindling in mice. Iran. J. Med. Sci. 41, 430–436. 27582593 PMC4967488

[B35] Liu J. Shi G. (2019). Pirfenidone activates cannabinoid receptor 2 in a mouse model of bleomycin-induced pulmonary fibrosis. Exp. Ther. Med. 18, 4241–4248. 10.3892/etm.2019.8045 31777533 PMC6862507

[B36] Liu J. Zhang P. Zou Q. Liang J. Chen Y. Cai Y. (2023). Status of epilepsy in the tropics: an overlooked perspective. Epilepsia Open 8, 32–45. 10.1002/EPI4.12686 36588194 PMC9977758

[B37] Liu N. Song Y. Liu T. Wang H. Yu N. Ma H. (2024). Metformin enhanced the effect of pirfenidone on pulmonary fibrosis in mice. Clin. Respir. J. 18, e13731. 10.1111/crj.13731 38286745 PMC10794892

[B38] Liu T. Zhang L. Joo D. Sun S.-C. (2017). NF-κB signaling in inflammation. Signal Transduct. Target Ther. 2, 17023. 10.1038/sigtrans.2017.23 29158945 PMC5661633

[B39] Löscher W. Potschka H. Sisodiya S. M. Vezzani A. (2020). Drug resistance in epilepsy: clinical impact, potential mechanisms, and new innovative treatment options. Pharmacol. Rev. 72, 606–638. 10.1124/pr.120.019539 32540959 PMC7300324

[B40] Lowry O. H. Rosebrough N. J. Farr A. L. Randall R. J. (1951). Protein measurement with the Folin phenol reagent. J. Biol. Chem. 1, 265–275. 10.1016/s0021-9258(19)52451-6 14907713

[B41] Madireddy S. Madireddy S. (2023). Therapeutic strategies to ameliorate neuronal damage in epilepsy by regulating oxidative stress, mitochondrial dysfunction, and neuroinflammation. Brain Sci. 13, 784. 10.3390/brainsci13050784 37239256 PMC10216584

[B42] Mir M. A. Malik A. B. Zulfkar D. Dar M. A. (2023). Adverse reactions caused by antiepileptic medications in real-world medical settings. Int. J. Curr. Res. Physiology Pharmacol., 25–35.

[B43] Misra H. P. Fridovich I. (1972). The role of superoxide anion in the autoxidation of epinephrine and a simple assay for superoxide dismutase. J. Biol. Chem. 247, 3170–3175. 10.1016/s0021-9258(19)45228-9 4623845

[B44] Misra H. P. Rabideau C. (2000). Pirfenidone inhibits NADPH-dependent microsomal lipid peroxidation and scavenges hydroxyl radicals. Mol. Cell Biochem. 204, 119–126. 10.1023/A:1007023532508 10718632

[B45] Nakamura Y. Shimizu Y. Fujimaki-Shiraishi M. Uchida N. Takemasa A. Niho S. (2023). A protective effect of pirfenidone in lung fibroblast–endothelial cell network via inhibition of rho-kinase activity. Biomedicines 11, 2259. 10.3390/biomedicines11082259 37626755 PMC10452915

[B46] Nodirahon A. Majid H. Waghdhare S. Vohora D. Nidhi (2024). The effect of sodium glucose Co-transport 2 inhibitors on cognitive impairment and depression in type 2 diabetes mellitus patients. Clin. Epidemiol. Glob. Health 26, 101555. 10.1016/J.CEGH.2024.101555

[B47] Novak A. Vizjak K. Rakusa M. (2022). Cognitive impairment in people with epilepsy. J. Clin. Med. 11, 267. 10.3390/jcm11010267 35012007 PMC8746065

[B48] Ohkawa H. Ohishi N. Yagi K. (1979). Assay for lipid peroxides in animal tissues by thiobarbituric acid reaction. Anal. Biochem. 95, 351–358. 10.1016/0003-2697(79)90738-3 36810

[B49] Palathingal Bava E. George J. Tarique M. Iyer S. Sahay P. Gomez Aguilar B. (2022). Pirfenidone increases IL-10 and improves acute pancreatitis in multiple clinically relevant murine models. JCI Insight 7, e141108. 10.1172/jci.insight.141108 34847076 PMC8855813

[B50] Pinto L. F. Silva L. S. João R. B. Boldrini V. Cendes F. Yasuda C. L. (2024). Practices in the prescription of antiseizure medications: is it time to change? Arq. Neuropsiquiatr. 82, 001–010. 10.1055/s-0043-1777806 PMC10965294 38531396

[B51] Ruwanpura S. M. Thomas B. J. Bardin P. G. (2020). Pirfenidone: molecular mechanisms and potential clinical applications in lung disease. Am. J. Respir. Cell Mol. Biol. 62, 413–422. 10.1165/rcmb.2019-0328TR 31967851

[B52] Salazar-Montes A. Ruiz-Corro L. López-Reyes A. Castrejón-Gómez E. Armendáriz-Borunda J. (2008). Potent antioxidant role of Pirfenidone in experimental cirrhosis. Eur. J. Pharmacol. 595, 69–77. 10.1016/j.ejphar.2008.06.110 18652820

[B53] Sehar N. Agarwal N. B. Vohora D. Raisuddin S. (2015). Atorvastatin prevents development of kindling by modulating hippocampal levels of dopamine, glutamate, and GABA in mice. Epilepsy and Behav. 42, 48–53. 10.1016/j.yebeh.2014.11.011 25499163

[B54] Sharawy M. H. Serrya M. S. (2020). Pirfenidone attenuates gentamicin-induced acute kidney injury by inhibiting inflammasome-dependent NLRP3 pathway in rats. Life Sci. 260, 118454. 10.1016/j.lfs.2020.118454 32950575

[B55] Soltani Khaboushan A. Yazdanpanah N. Rezaei N. (2022). Neuroinflammation and proinflammatory cytokines in epileptogenesis. Mol. Neurobiol. 59, 1724–1743. 10.1007/s12035-022-02725-6 35015252

[B56] Song Z. Wu T. Sun J. Wang H. Hua F. Nicolas Y. S. M. (2021). Metformin attenuates post‐epidural fibrosis by inhibiting the TGF‐β1/Smad3 and HMGB1/TLR4 signaling pathways. J. Cell Mol. Med. 25, 3272–3283. 10.1111/jcmm.16398 33611840 PMC8034438

[B57] Stasenko A. Lin C. Bonilha L. Bernhardt B. C. McDonald C. R. (2024). Neurobehavioral and clinical comorbidities in epilepsy: the role of white matter network disruption. Neurosci. 30, 105–131. 10.1177/10738584221076133 PMC9393207 35193421

[B58] Suleymanova E. M. (2021). Behavioral comorbidities of epilepsy and neuroinflammation: evidence from experimental and clinical studies. Epilepsy and Behav. 117, 107869. 10.1016/j.yebeh.2021.107869 33684786

[B59] Tavakoli Z. Tahmasebi Dehkordi H. Lorigooini Z. Rahimi-Madiseh M. Korani M. S. Amini-Khoei H. (2023). Anticonvulsant effect of quercetin in pentylenetetrazole (PTZ)-induced seizures in male mice: the role of anti-neuroinflammatory and anti-oxidative stress. Int. Immunopharmacol. 116, 109772. 10.1016/j.intimp.2023.109772 36731152

[B60] Tian Q. Xiao Q. Yu W. Gu M. Zhao N. Lü Y. (2016). The inhibition of transforming growth factor beta-activated kinase 1 contributed to neuroprotection via inflammatory reaction in pilocarpine-induced rats with epilepsy. Neuroscience 325, 111–123. 10.1016/J.NEUROSCIENCE.2016.03.045 27012613

[B61] Tombini M. Boscarino M. Di Lazzaro V. (2023). Tackling seizures in patients with Alzheimer’s disease. Expert Rev. Neurother. 23, 1131–1145. 10.1080/14737175.2023.2278487 37946507

[B62] Tyagi K. Masoom M. Majid H. Garg A. Bhurani D. Agarwal N. B. (2023). Role of cytokines in chemotherapy-related cognitive impairment of breast cancer patients: a systematic review. Curr. Rev. Clin. Exp. Pharmacol. 18, 110–119. 10.2174/2772432817666220304212456 35249524

[B63] Ueda Y. Doi T. Tokumaru J. Yokoyama H. Nakajima A. Mitsuyama Y. (2001). Collapse of extracellular glutamate regulation during epileptogenesis: down‐regulation and functional failure of glutamate transporter function in rats with chronic seizures induced by kainic acid. J. Neurochem. 76, 892–900. 10.1046/j.1471-4159.2001.00087.x 11158261

[B64] Vezzani A. Balosso S. Ravizza T. (2008). The role of cytokines in the pathophysiology of epilepsy. Brain Behav. Immun. 22, 797–803. 10.1016/J.BBI.2008.03.009 18495419

[B65] Vidaurre J. Gedela S. Yarosz S. (2017). Antiepileptic drugs and liver disease. Pediatr. Neurol. 77, 23–36. 10.1016/J.PEDIATRNEUROL.2017.09.013 29097018

[B66] Walker J. E. Giri S. N. Margolin S. B. (2005). A double-blind, randomized, controlled study of oral pirfenidone for treatment of secondary progressive multiple sclerosis. Multiple Scler. J. 11, 149–158. 10.1191/1352458505ms1134oa 15794387

[B67] Wang F. Wen T. Chen X. Y. Wu H. (2008). Protective effects of pirfenidone on D-galactosamine and lipopolysaccharide-induced acute hepatotoxicity in rats. Inflamm. Res. 57, 183–188. 10.1007/S00011-007-7153-8 18344059

[B68] Wang Y. Wang G. Tao J. Li X. Hu L. Li Q. (2020). Autophagy associated with the efficacy of valproic acid in PTZ-induced epileptic rats. Brain Res. 1745, 146923. 10.1016/j.brainres.2020.146923 32504548

[B69] Waris A. Siraj M. Khan A. Lin J. Asim M. Alhumaydh F. A. (2024). A comprehensive overview of the current status and advancements in various treatment strategies against epilepsy. ACS Pharmacol. Transl. Sci. 7, 3729–3757. 10.1021/acsptsci.4c00494 39698272 PMC11650742

[B70] Wu J. Cai Y. Wu X. Ying Y. Tai Y. He M. (2021). Transcardiac perfusion of the mouse for brain tissue dissection and fixation. Bio Protoc. 11, e3988. 10.21769/BioProtoc.3988 PMC8005872 33796622

[B71] Xiao M. Qu X. H. Lv J. P. Shi Y. Li C. X. Xie K. J. (2016). Effects of pirfenidone on hepatic fibrosis in mice induced by carbon tetrachloride. Zhongguo Ying Yong Sheng Li Xue Za Zhi 32 (4), 378–382. 10.13459/J.CNKI.CJAP.2016.04.023 29931966

[B72] Yan X. Jiang E. Weng H.-R. (2015). Activation of toll like receptor 4 attenuates GABA synthesis and postsynaptic GABA receptor activities in the spinal dorsal horn via releasing interleukin-1 beta. J. Neuroinflammation 12, 222. 10.1186/s12974-014-0222-3 25571780 PMC4302431

[B73] Zhang H. Zhang R. Chen J. Shi M. Li W. Zhang X. (2017). High mobility group Box1 inhibitor glycyrrhizic acid attenuates kidney injury in streptozotocin-induced diabetic rats. Kidney Blood Press Res. 42, 894–904. 10.1159/000485045 29241180

[B74] Zhao M. Zhou A. Xu L. Zhang X. (2014). The role of TLR4-mediated PTEN/PI3K/AKT/NF-κB signaling pathway in neuroinflammation in hippocampal neurons. Neuroscience 269, 93–101. 10.1016/J.NEUROSCIENCE.2014.03.039 24680857

[B75] Zhao X. Cheng P. Xu R. Meng K. Liao S. Jia P. (2022). Insights into the development of pentylenetetrazole-induced epileptic seizures from dynamic metabolomic changes. Metab. Brain Dis. 37, 2441–2455. 10.1007/s11011-022-01018-0 35838870

[B76] Zhong R. Chen Q. Li M. Zhang X. Lin W. (2019). Elevated blood C-reactive protein levels in patients with epilepsy: a systematic review and meta-analysis. Front. Neurol. 10, 974. 10.3389/fneur.2019.00974 31620066 PMC6759543

[B77] Zhou X. Zhang C. Wang L. Jin S. (2022). Remimazolam induced cognitive dysfunction in mice via glutamate excitotoxicity. Transl. Neurosci. 13, 104–115. 10.1515/tnsci-2022-0220 35734308 PMC9164290

[B78] Zhu L. Chen L. Xu P. Lu D. Dai S. Zhong L. (2020). Genetic and molecular basis of epilepsy-related cognitive dysfunction. Epilepsy and Behav. 104, 106848. 10.1016/j.yebeh.2019.106848 32028124

[B79] Zou J. Crews F. T. (2015). Glutamate/NMDA excitotoxicity and HMGB1/TLR4 neuroimmune toxicity converge as components of neurodegeneration. AIMS Mol. Sci. 2, 77–100. 10.3934/molsci.2015.2.77

